# Beyond Urease: New Potential Enzymatic Targets in *Helicobacter pylori*

**DOI:** 10.1021/acsomega.6c00763

**Published:** 2026-06-04

**Authors:** Ana Micaela Camini, Luiza Rosa Cogo, Maria Eduarda Delawi, Débora Bublitz Anton, Juliana Koakovski Acosta, Jeferson Camargo de Lima, Luís Fernando Saraiva Macedo Timmers

**Affiliations:** † 186081 Universidade do Vale do Taquari Univates, Lajeado, Rio Grande do Sul 95914-014, Brazil; ‡ Programa de Pós-Graduação em Biotecnologia (PPGBiotec), Univates, Lajeado, Rio Grande do Sul 95914-014, Brazil; § Programa de Pós-Graduação em Ciências Médicas (PPGCM), Univates, Lajeado, Rio Grande do Sul 95914-014, Brazil

## Abstract

*H. pylori* infection remains one of the most widespread bacterial diseases globally and a leading risk factor for peptic ulcer disease and gastric cancer. Despite decades of research, the treatment of *H. pylori* still depends on multidrug antibiotic regimens, whose efficacy is waning due to the rise in antimicrobial resistance. Although significant progress has been made in understanding *H. pylori* pathogenesis, most studies targeting bacterial enzymes have focused almost exclusively on urease, a well-characterized virulence factor, while other metabolic and structural pathways remain comparatively underexplored. Targeting essential enzymes involved in bacterial metabolism and structural integrity may disrupt vital processes, potentially reducing off-target effects on the host microbiota and overcoming resistance mechanisms. To address this gap, this systematic review, conducted according to PRISMA 2020 guidelines, synthesized experimental studies published between 2014 and 2024 that investigated enzyme-targeted compounds against *H. pylori*, excluding those focused on urease. Literature searches in PubMed and the Web of Science identified 49 eligible studies exploring enzymes across multiple metabolic pathways. The main pathways identified included purine metabolism, the shikimate and futalosine pathways, and nitrogen metabolism, along with several other enzymatic systems, such as thioredoxin, thymidylate, and peptidoglycan biosynthesis, all of which represent promising targets for selective inhibition in *H. pylori*. The reported inhibitors exhibited micromolar to submicromolar activity and, in some cases, demonstrated potent antibacterial effects with minimal cytotoxicity. However, most studies remained limited to *in vitro* assays, and only three included animal tests. These findings highlight enzymatic inhibition as a promising approach for the rational design of narrow-spectrum microbiome-sparing agents. Advancing these discoveries through *in vivo* validation and druggability assessment will be essential to translating enzyme-based inhibition into effective therapeutic options against *H. pylori*.

## Graphical abstract

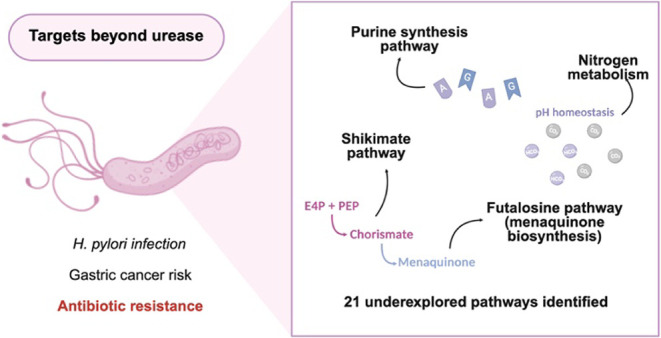

## 1 Introduction

*Helicobacter pylori* (*H. pylori*) is a Gram-negative, microaerophilic bacterium that colonizes the human stomach and is typically transmitted during childhood, persisting throughout life if untreated.^1–3^ It currently infects about 43% of the global population, making it one of the most widespread bacterial infections in the world. Prevalence varies considerably depending on geographic regions and sanitation conditions, reaching much higher levels in developing countries.^4^ The diverse pathologies attributed to *H. pylori* infection result from complex interactions among bacterial virulence factors, host genetic susceptibility, and environmental influences, leading to different clinical phenotypes of chronic gastritis.^5–7^ These mechanisms highlight potential therapeutic vulnerabilities, as interfering with these pathways could impair bacterial survival and limit disease progression.^6–8^

Persistent *H. pylori* infection is a major risk factor for serious gastroduodenal diseases, including peptic ulcers and gastric adenocarcinoma. Notably, *H. pylori* is estimated to be responsible for approximately 89% of gastric cancer cases, a disease that ranks as the fifth most common cancer worldwide and the fifth leading cause of cancer-related deaths.^9,10^ Because of its significant global health impact, *H. pylori* is classified as a Class 1 carcinogen by the World Health Organization (WHO) and the International Agency for Research on Cancer (IARC).^11^ Eradication of *H. pylori* not only promotes healing of gastric mucosal damage but also markedly reduces the long-term risk of developing gastric malignancies, ulcerative disease, and MALT lymphoma, underscoring the importance of effective treatment strategies.^12,13^

Current treatment strategies for *H. pylori* infection rely on multidrug regimens that combine antibiotics, such as clarithromycin, amoxicillin, metronidazole, tetracycline, or levofloxacin, with proton pump inhibitors (PPIs) to suppress gastric acid secretion and enhance the eradication rate.^14–16^ However, increasing antibiotic resistance, particularly to clarithromycin, metronidazole, and levofloxacin, has substantially reduced treatment efficacy worldwide, with eradication failure rates frequently exceeding 20–30% in some regions.^14–18^ Recent international consensus guidelines, including Maastricht VI/Florence (2022) and the American College of Gastroenterology (2024), now recommend bismuth-containing quadruple therapy (BQT) as the preferred first-line regimen in most areas with moderate to high resistance or unknown susceptibility profiles.^16,19^ Furthermore, the antibacterial development pipeline shows a critical lack of novel agents specifically targeting *H. pylori*, representing a major barrier to therapeutic innovation against this priority pathogen.^20,21^

Despite advances in understanding the pathogenesis and treatment of *Helicobacter pylori*, significant challenges remain.^6^ The bacterium’s localization within deep gastric microniches and the difficulties associated with its *in vitro* cultivation complicate both drug delivery and the development of narrow-spectrum therapeutics. Moreover, incomplete knowledge of resistance mechanisms and immune evasion continues to hinder innovation.^22,23^ Among the enzymatic systems characterized in *H. pylori*, urease remains the most extensively studied, reflecting its critical role in acid neutralization and gastric colonization.^24,25^ Consequently, most available studies and inhibitor designs focus on this enzyme, whereas other essential metabolic and structural pathways remain comparatively underexplored. Expanding research toward these alternative enzymatic pathways is crucial for identifying new therapeutic vulnerabilities. Enzyme inhibition thus represents a promising strategy to disrupt vital bacterial processes, minimize off-target effects on the host microbiota, and help overcome antibiotic resistance.^26,27^

To address this gap, the present systematic review compiles experimental studies published between 2014 and 2024 that investigated enzyme-targeted compounds against *H. pylori*, focusing specifically on nonurease enzymes. The data were systematically collected and organized to identify the main enzymatic pathways explored and their biological relevance to the bacterial viability. By integrating these findings, this work aims to highlight enzymes that remain underexplored in *H. pylori* as potential therapeutic targets and to guide future directions for drug development against this pathogen.

## 2 Methodology

### 2.1 Literature Search

This systematic review was conducted in accordance with the PRISMA 2020 (Preferred Reporting Items for Systematic Reviews and Meta-Analyses) guidelines.^28^ Literature searches were performed in MEDLINE (via PubMed) and Web of Science (via Clarivate) to identify studies published between January 1, 2014, and December 31, 2024.

In PubMed, the search strategy included the following Medical Subject Headings (MeSH) terms: (“*Helicobacter pylori*”[Mesh]) AND (“enzyme inhibitors”[Mesh]). Filters were applied to select only original studies, including classical experimental articles, comparative studies, and clinical trials, while excluding nonoriginal research. Additionally, studies supported by various funding sources (e.g., Research Support, American Recovery and Reinvestment Act; Research Support, N.I.H., Extramural; Research Support, N.I.H., Intramural; Research Support, Non-U.S. Gov’t; Research Support, U.S. Gov’t; Research Support, U.S. Gov’t, Non-P.H.S.; Research Support, U.S. Gov’t, P.H.S.) were included.

In Web of Science, a free-text search using the keywords *“* *Helicobacter pylori*” AND “enzyme inhibitors” was conducted. The “Article” filter was applied to select only primary research studies for inclusion in the final selection.

### 2.2 Eligibility Criteria

To minimize selection bias, inclusion and exclusion criteria were established prior to the literature search. The retrieved articles were required to meet the following inclusion criteria: (1) articles published in English and (2) original experimental research articles with data on the development or testing of compounds targeting specific *H. pylori* enzymes. Clinical studies and comparative studies were not excluded at this stage, but only those that explicitly identified and tested compounds targeting specific *H. pylori* pathways (via *in vitro* experiments on enzymes or *in vivo* testing with *H. pylori* culture) were selected for inclusion. Studies were excluded if they met any of the following criteria: (1) based solely on computational data; (2) investigated compounds that did not target a specific *H. pylori* pathway but only mitigated infection-related effects (e.g., reducing inflammation); (3) were clinical studies that did not specify the pathway targeted by the compounds; (4) were case reports, reviews, systematic reviews, conference abstracts, or unpublished papers; or (5) focused exclusively on the urease enzyme.

### 2.3 Study Selection Process

Literature screening was carried out by two independent reviewers (A.M.C. and L.R.C.) in three separate stages of screening: title, abstract, and then full text. Each reviewer compiled a data sheet documenting whether an article should be included or excluded, including reasons for exclusion. Disagreements were resolved through independent review by a third reviewer (J.K.A.), and any unresolved discrepancies were discussed as a team. All retrieved articles were screened for relevance on the basis of predefined inclusion and exclusion criteria.

### 2.4 Data Extraction

Two reviewers (A.M.C. and L.R.C.) independently extracted data using structured spreadsheets designed for standardized data collection. Reviewers worked independently to ensure unbiased extraction and did not access each other’s files until completion. Extracted variables included study identifiers, the targeted enzyme or pathway, compound identification, enzymatic inhibition parameters such as the inhibition constant (K_i_); antibacterial activity such as the half-maximal inhibitory concentration (IC_50_), minimum inhibitory concentration (MIC), and minimum bactericidal concentration (MBC); cytotoxicity data (cell line and assay type); and animal model outcomes.

After extraction, both data sets were compared for consistency. Any discrepancies were resolved through joint reevaluation of the original articles, and the verified information was consolidated into a single summary table. Reported values were standardized to a common unit of measurement, when applicable, to improve clarity and ensure a uniform presentation across studies.

### 2.5 Synthesis and Analysis of Results

The synthesis focused on describing the biological pathways and enzymes targeted by the reported inhibitors, emphasizing the experimental methodologies used (*in vitro*, *in vivo*, or hybrid approaches). The analysis was qualitative, comparing studies based on the targeted pathways and enzymes, evaluating their biological and clinical relevance, and identifying the current research gaps. Variations in experimental methodology and observed outcomes are also discussed in relation to the biological significance of each enzyme.

## 3 Results

### 3.1 Study Selection

A total of 1755 records were initially identified through database searches, comprising 1245 records from PubMed and 510 from the Web of Science. After removal of reviews and application of predefined filters, 517 records from PubMed and 399 from the Web of Science remained. Subsequently, 25 PubMed articles without abstracts were excluded, leaving 492 PubMed and 399 Web of Science articles.

A total of 65 duplicates were identified and removed, resulting in 826 unique abstracts for screening. Upon screening of titles and abstracts against the inclusion criteria, 757 records were excluded for not meeting the eligibility criteria. A total of 69 full-text articles were assessed in detail. A total of 17 articles were excluded for reasons including lack of methodological rigor or population mismatch, and 3 were inaccessible due to full-text availability restrictions. Ultimately, 49 studies met all inclusion criteria and were included in this Systematic Review. A PRISMA flow diagram illustrating the selection process is presented in Figure 1.

**Figure 1:**
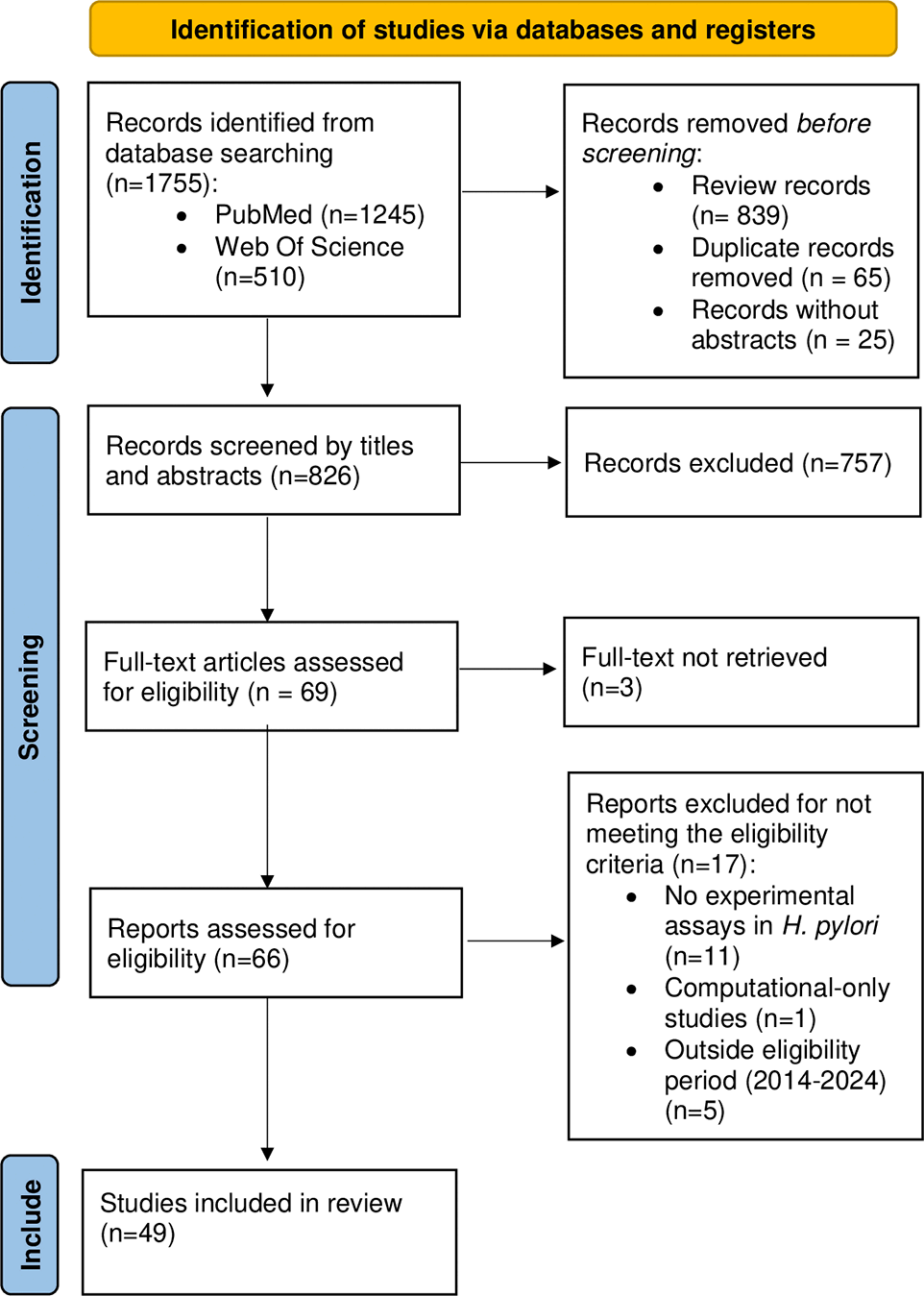
PRISMA 2020 flow of information through the study selection phases. Adapted from Page et al.,^28^ distributed under a Creative Commons CC BY 4.0 license.

### 3.2 Inhibition Data and Experimental Approaches

The studies included in this review exhibited considerable variability in their experimental approaches and in the types of inhibition data reported. Several publications presented enzymatic characterizations using parameters such as the inhibition constant (K_i_), half-maximal inhibitory concentration (IC_50_), and mode of inhibition. In contrast, other studies reported inhibition as percentage values obtained at single fixed concentrations, rather than from complete dose–response curves or kinetic analyses. A subset of studies reported both enzymatic inhibition and antibacterial activity, such as Minimum Inhibitory Concentration (MIC) and Minimum Bactericidal Concentration (MBC) measurements. This adds translational value, but in some articles the strain information was absent (Table S1).

Cytotoxicity assessments were inconsistently performed; some included quantitative data (LC_50_ or viability), while others lacked any toxicity evaluation. Only three studies extended testing to animal models, none providing pharmacokinetic or bioavailability data, thus limiting translational interpretation (Table S2).

In studies that evaluated larger sets of compounds, the five most promising candidates, based on bacterial growth inhibition and enzymatic inhibition parameters reported in each study, were extracted and summarized in Tables S1 and S2 to standardize data presentation and facilitate comparison across heterogeneous experimental designs.

### 3.3 Potential Therapeutic Target Pathways in *H. pylori*

The 49 included studies reported multiple enzymatic targets in *H. pylori* (Figure 2). These enzymes were grouped according to their respective metabolic pathways or cellular processes, as individual studies often investigated more than one target (Table 1). More than half of the studies focused on enzymes involved in purine metabolism, menaquinone biosynthesis, the shikimate pathway, and nitrogen metabolism, whereas the remaining pathways were represented by only one or two articles. This distribution highlights the predominance of research within the four metabolic routes. Because several studies evaluated large numbers of compounds, a standardized approach was adopted in which only the five inhibitors with the lowest enzymatic or antibacterial values reported in each study were extracted and summarized in Table S1. The descriptive results presented in the following subsections refer to the standardized subset. The detailed findings for each of these four predominant pathways are presented in the subsections below, followed by a synthesis of the additional underexplored targets identified in the remaining studies.

**Figure 2:**
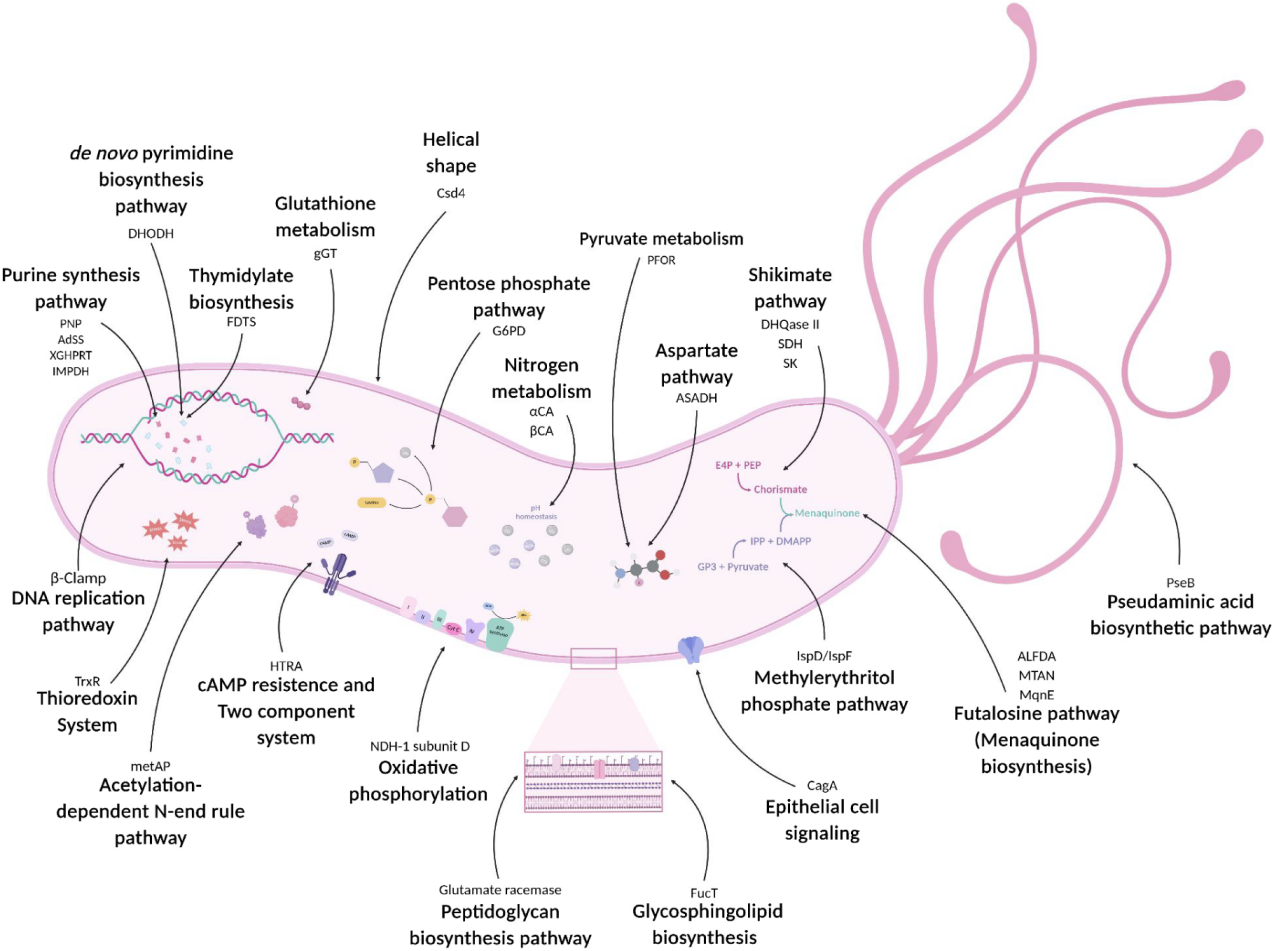
Overview of enzymatic pathways explored as therapeutic targets in *H. pylori*. PNP, purine nucleoside phosphorylase; AdSS, adenylosuccinate synthetase; XGHPRT, xanthine-guanine-hypoxanthine phosphoribosyltransferase; IMPDH, inosine-5′-monophosphate dehydrogenase; SK, shikimate kinase; SDH, shikimate dehydrogenase; DHQase III, 3-dehydroquinase dehydratase type II; MTAN, aminodeoxyfutalosine nucleosidase; AFLDA, aminofutalosine deaminase; MqnE, aminodeoxyfutalosine/aminofutalosine synthase; αCA and βCA, carbonic anhydrases α and β; glutamate racemase; NDH-1 subunit D, NADH-quinone oxidoreductase subunit D; IspD/IspF, bifunctional enzyme IspD/IspF; DHODH, dihydroorotate dehydrogenase; UDP-N-acetylglucosamine 4,6-dehydratase; β-Clamp, β sliding clamp; TrxR, thioredoxin reductase; FDTS, flavin-dependent thymidylate synthase; HTRA, high-temperature requirement A serine protease; Csd4, DL-carboxypeptidase; CagA, cytotoxin-associated pathogenicity island protein 1; gGT, glutathione hydrolase/γ-glutamyltranspeptidase; G6PD, glucose-6-phosphate 1-dehydrogenase; metAP, methionine/methionyl aminopeptidase; ASADH, aspartate-semialdehyde dehydrogenase; FucT, α-1,3-fucosyltransferase; PFOR, pyruvate ferredoxin oxidoreductase.

**Table 1:** Enzymatic Targets Included in This Systematic Review^a^

Pathway	Gene	Protein	Human homologue	Number of studies
Purine synthesis pathway	*deoD*	Purine nucleoside phosphorylase	Yes	4
	*purA*	Adenylosuccinate synthetase	Yes	3
	*gpt*	Xanthine-guanine-hypoxanthine phosphoribosyltransferase	Yes	1
	*guaB*	Inosine-5′-monophosphate dehydrogenase	Yes	7
Shikimate pathway	*aroK*	Shikimate kinase	No	4
	*aroE*	Shikimate dehydrogenase	No	1
	*aroQ*	3-dehydroquinase dehydratase type II	No	1
Futalosine pathway (menaquinone biosynthesis)	*mtnN*	Aminodeoxyfutalosine nucleosidase	No	4
	*mqnF*	Aminodeoxyfutalosine deaminase	No	1
	*mqnE*	Aminodeoxyfutalosine synthase	No	1
Nitrogen Metabolism	*cynT*	Carbonic anhydrases α and β	Yes	5
Peptidoglycan biosynthesis pathway	*murI*	Glutamate racemase	No	2
Oxidative phosphorylation	*nuoD*	NADH-quinone oxidoreductase subunit D	Yes	1
Methylerythritol phosphate pathway (MEP pathway)	*ispDF*	Bifunctional enzyme IspD/IspF	No	2
*de novo* pyrimidine biosynthesis pathway	*pyrD*	Dihydroorotate dehydrogenase	Yes	1
Pseudaminic acid biosynthetic pathway	*pseB*	UDP-N-acetylglucosamine 4,6-dehydratase	No	1
DNA replication pathway	*dnaN*	β sliding clamp	No	1
Thioredoxin system	*trxB*	Thioredoxin reductase	Yes	2
Thymidylate biosynthesis	*thyX*	Flavin-dependent thymidylate synthase	No	1
cAMP resistance and two component system	*htrA*	High-temperature requirement A serine protease	Yes	1
Helical shape	*csd4*	DL-carboxypeptidase	No	1
Epithelial cell signaling	*cagA*	Cytotoxin-associated pathogenicity island protein 1	No	2
Glutathione metabolism	*ggt*	Glutathione hydrolase/γ-glutamyltranspeptidase	Yes	1
Pentose phosphate pathway	*zwf*	Glucose-6-phosphate 1-dehydrogenase	Yes	1
Acetylation-dependent N-end rule pathway	*map*	Methionine/methionyl aminopeptidase	Yes	1
Aspartate pathway	*asd*	Aspartate-semialdehyde dehydrogenase	No	1
Glycosphingolipid biosynthesis	*fucT*	α-1,3-fucosyltransferase	Yes	1
Pyruvate metabolism	*porA (subunit α), porB (subunit β), porC (subunit γ), and porD (subunit δ) (different subunits of the PFOR complex)*	Pyruvate ferredoxin oxidoreductase	No	1

a Data were compiled from public enzyme databases: KEGG,^29^ UniProt,^30^ and BRENDA.^31^

The detailed findings for each of these four predominant pathways are presented in the subsections below, followed by a synthesis of additional underexplored targets identified in the remaining studies. Additional structural and binding-related information reported across studies is compiled in Table S3, limited to data available in the studies included in this Review.

#### 3.3.1 Purine Metabolism

Purine metabolism was one of the pathways that were most frequently investigated across the included studies. This pathway supplies the nucleotides required for DNA and RNA synthesis and for essential cellular functions such as energy transfer, signaling, and cofactor generation.^32^ Most organisms, including humans, possess both *de novo* and salvage routes for purine nucleotide production. However, genomic analyses have shown that *H. pylori* retains only the salvage pathway; consequently, enzymes from this route were recurrently evaluated among the included studies.^33^

Four enzymes associated with this pathway were examined. These were purine nucleoside phosphorylase (PNP), adenylosuccinate synthetase (AdSS), xanthine-guanine-hypoxanthine phosphoribosyltransferase (XGHPRT), and inosine monophosphate dehydrogenase (IMPDH). PNP, AdSS, and XGHPRT participate directly in the salvage route.^34^ Meanwhile, IMPDH is involved in a metabolic branch-point reaction in the synthesis of guanine nucleotides, and in *H. pylori*, its activity depends on IMP generated through *salvage* reactions (Figure 3a).

**Figure 3:**
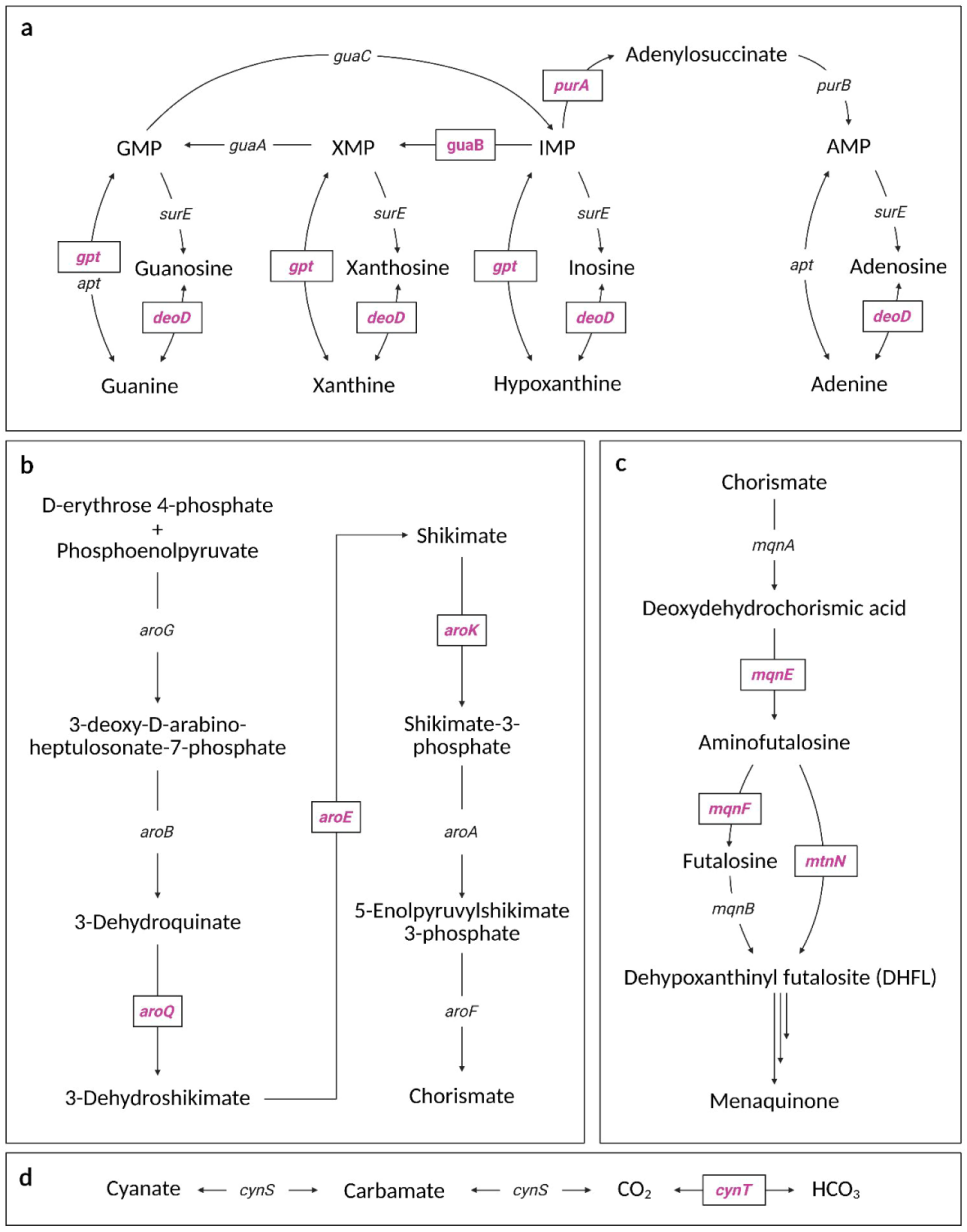
Most extensively studied metabolic pathways in *Helicobacter pylori*, beyond urease. (a) Purine synthesis pathway; (b) shikimate pathway; (c) futalosine pathway (menaquinone biosynthesis); (d) nitrogen metabolism (only the conversion of cyanate to bicarbonate is represented). Genes corresponding to the enzymes identified in this review are shown in magenta within rounded boxes. Gene abbreviations are followed by the enzyme they encode: *guaA*, guanosine monophosphate synthase; *guaB*, inosine-5′-monophosphate dehydrogenase; *guaC*, guanosine monophosphate reductase; *purA*, adenylosuccinate synthetase; *purB*, adenylosuccinate lyase; *surE*, 5′-nucleotidase SurE; *deoD*, purine nucleoside phosphorylase; *gpt*, xanthine-guanine-hypoxanthine phosphoribosyltransferase; *apt,* adenine phosphoribosyltransferase; *aroG*, 3-deoxy-D-arabino-heptulosonate 7-phosphate synthase; *aroB*, dehydroquinate synthase; *aroQ*, dehydroquinase type II; *aroE*, shikimate dehydrogenase; *aroK*, shikimate kinase; *aroA*, 5-enolpyruvylshikimate-3-phosphate synthase; *aroF*, chorismate synthase; *mqnA*, chorismate dehydratase; *mqnE*, aminodeoxyfutalosine/aminofutalosine synthase; *mqnF*, aminofutalosine deaminase; *mqnB*, futalosine hydrolase; *mtnN*, aminodeoxyfutalosine nucleosidase; *cynS*, cyanate hydratase; and *cynT*, α- and β-carbonic anhydrases.

Across the three studies evaluating PNP, one investigated purine analogs substituted at positions 2 and 6. These analogs inhibited the enzyme with low-micromolar K_i_ values and reduced *H. pylori* growth in culture. Among those tested, compounds containing a benzylthio substituent at position 6 showed the lowest K_i_ values.^35^ The other two studies examined the known PNP inhibitors formycin A/B and described their binding modes based on structural analyses of the enzyme–ligand complexes, reporting interactions within the active site that were consistent with their inhibitory activity.^36,37^ Regarding AdSS, hadacidin displayed competitive inhibition with K_i_ values down to 0.19 μM and demonstrated antibacterial activity.^38^ Pyridoxal 5′-phosphate (PLP) was also evaluated as an AdSS inhibitor, showing higher K_i_ values than hadacidin but lower MIC values, ranging from 185 to 309 μM against clinical *H. pylori* strains.^39^

For XGHPRT, the reported inhibitors exhibited K_i_ values between 0.2 and 5 μM and inhibited *H. pylori* growth when tested at 50 μM, with negligible cytotoxicity based on previously reported compound data.^40^ In the case of IMPDH, both natural and synthetic molecules demonstrated high selectivity over the human isoform (IC_50_ = 0.095–10.7 μM) and antibacterial activity with MIC values as low as 0.98 μg/mL.^41–47^ One study also evaluated a combination of amoxicillin with compound 6 at a 2:1 ratio, which showed an IC_50_ value of 0.59 μg/mL and was more active than the reference drugs. This compound also showed selectivity over the human IMPDH2 enzyme, with IC_50_ values greater than 10 μg/mL. *In silico* ADMET predictions also suggested a favorable pharmacokinetic profile comparable to standard antibiotics.^42^

#### 3.3.2 Shikimate Pathway

The shikimate pathway, which is essential for the biosynthesis of chorismate and downstream aromatic metabolites, plays a central role in the production of aromatic amino acids, folate intermediates, and menaquinone precursors in *H. pylori*. Because this critical pathway is absent in humans, its enzymes represent attractive targets for selective therapeutic intervention.^48^ In the studies included in this Review, three enzymes belonging to this pathway were investigated: shikimate kinase (SK), shikimate dehydrogenase (SDH), and 3-dehydroquinase dehydratase type II (DHQase II) (Figure 3b).

Most studies investigated SK, reporting low micromolar inhibition (K_i_ = 0.30–15.5 μM) and focusing primarily on structural and kinetic analyses.^46–51^ Two studies also evaluated antibacterial activity, with reported MIC values ranging from 4 to 800 μg/mL, the lowest value reported of which was 4 μg/mL.^49,52^ The single study on SDH assessed antibacterial activity rather than enzymatic inhibition. A virtual screening protocol was first used to identify compounds predicted to interact with the SDH active site, and subsequent experimental testing showed that 11 of these compounds had MIC values ranging from 8 to 93 μg/mL against two *H. pylori* reference strains.^53^ Finally, for DHQase II, K_i_ values ranged from 6.3 to 85 μM, and the study focused on structural characterization and ligand fragment optimization.^54^

#### 3.3.3 Futalosine Pathway

The futalosine pathway is the alternative route for menaquinone biosynthesis in *H. pylori*, which relies exclusively on this pathway for menaquinone production.^55^ Crucially, this pathway is absent in humans and is essential for bacterial respiration, making its enzymes critical for *H. pylori* survival.^56^ Three enzymes belonging to this pathway were reported in the included studies: aminodeoxyfutalosine nucleosidase (MTAN), aminofutalosine deaminase (AFLDA), and aminodeoxyfutalosine/aminofutalosine synthase (MqnE) (Figure 3C).

MTAN was evaluated in several studies, which described inhibitors including DADMe- and Immucillin-based analogs, with K_i_ values in the nanomolar range (down to 0.00003 μM).^57–60^ Although HT-DADMe-ImmA was initially developed as an MTAN inhibitor based on studies in *Mycobacterium tuberculosis*, the nonessentiality of this enzyme in that pathogen redirected investigations toward *H. pylori*. In this context, HT-DADMe-ImmA exhibited remarkable potency, inhibiting bacterial growth with an IC_50_ of 13.0 ± 1.8 ng/mL, approximately six times more effective than tetracycline.^58^ Other MTAN-targeting analogs also demonstrated strong antibacterial effects, with IC_90_ values reaching as low as 8 ng/mL in *H. pylori* cultures.^59^

Regarding AFLDA, inhibitors exhibited K_i_ values between 0.063 and 0.85 μM, and the most active compound showed an IC_50_ of 14 μM in *H. pylori* culture.^61^ Finally, for MqnE, the methylene analog 9 was identified as an inhibitor and showed antibacterial activity with an IC_50_ of 1.8 ± 0.4 μM in bacterial culture.^62^

#### 3.3.4 Nitrogen Metabolism

Nitrogen metabolism in *H. pylori* encompasses several processes required for acid acclimation and intracellular pH control, including reactions that interconvert nitrogen- and carbon-containing species to support survival in the gastric environment.^63^ Within this context, α- and β-carbonic anhydrases play a central role by catalyzing the reversible hydration of carbon dioxide to bicarbonate and protons, thereby supplying bicarbonate for biosynthetic pathways and contributing to the buffering system that operates alongside urease (Figure 3d).^64,65^

Five studies investigating *H. pylori* carbonic anhydrases were included in this review; four of which focused exclusively on the α-isoform, while one study analyzed both α- and β-isoforms. Reported inhibitory constants (K_i_) ranged from 0.02 to >100 μM and MIC from 8 to 128 μg/mL. None of the studies directly assessed cytotoxicity, although one referenced previous evidence of nontoxicity in AGS gastric cells. Sulfonamide derivatives were reported as inhibitors in the low-micromolar to submicromolar range.^66–70^

#### 3.3.5 Additional Potential Therapeutic Targets

Our systematic review identified several underexplored molecular targets in *H. pylori*. These enzymes span pathways such as peptidoglycan biosynthesis,^71,72^ oxidative phosphorylation,^73^ the methylerythritol phosphate (MEP) pathway,^74,75^ *de novo* pyrimidine biosynthesis,^76^ aspartate and pseudaminic acid metabolism (Pse),^77^ DNA replication,^78^ the thioredoxin system,^79,80^ and thymidylate biosynthesis,^81^ among others.^82–89^ Notably, these targets were reported in only one or two studies (Table S1).

Among all the enzymes identified in this systematic review, only glutamate racemase, flavin-dependent thymidylate synthase X (FDTS), and dihydroorotate dehydrogenase (DHODH) were evaluated in animal models.^71,76,81^ Glutamate racemase inhibitors exhibited antibacterial activity against clinical isolates (MIC = 0.13–64 μg/mL) but failed to reduce bacterial burden in infected mice even at the highest tested dose (100 mg/kg/day).^71^ FDTS inhibitors, including compounds 010-E, 010-C, and 010-I, presented selective inhibition (K_i_ = 0.028–1 μM) and low cytotoxicity (>50 μg/mL in AGS cells), with compound 010-I producing a 17-fold reduction in bacterial load *in vivo*.^81^ DHODH inhibitors showed enzymatic inhibition with IC_50_ values ranging from 0.06 to 11.1 μM and antibacterial activity with MIC values between 0.0125 and 1 μg/mL. Among them, compound AS1934 displayed stronger anti-*H. pylori* activity than AS1664 and reduced bacterial counts in infected mice; treated animals showed no significant body weight loss, indicating low adverse-effect occurrence.^76^

The additional enzymes summarized in Table 1, together with the compound-related data presented in Tables S1–S3, provide biochemical, structural, and functional information that expands the characterization of *H. pylori* metabolic pathways and illustrates the diversity of molecular targets reported across the included studies.

## 4 Discussion

Gastric cancer has a multifactorial etiology in which *Helicobacter pylori* is recognized as the primary microbial driver of gastric carcinogenesis.^9,10^ Although many infections remain asymptomatic, the bacterium is strongly associated with chronic gastritis, peptic ulcer disease, mucosa-associated lymphoid tissue lymphoma, and noncardiac gastric adenocarcinoma.^90^ Consequently, eradication therapy remains a central strategy for preventing long-term gastric complications and reducing the global gastric cancer burden.^12,13^

Standard clinical practice for *H. pylori* eradication relies on combinations of proton pump inhibitors and broad-spectrum antibiotics, typically administered as triple or quadruple therapies and often supplemented with bismuth salts.^14–16^ These agents act on highly conserved bacterial processes such as cell wall biosynthesis (amoxicillin),^91^ protein synthesis (clarithromycin, tetracycline),^92^ DNA damage (metronidazole, furazolidone),^93,94^ RNA polymerase inhibition (rifabutin),^95^ and replication interference (levofloxacin).^96,97^ Although initially effective, their clinical success has been markedly compromised by the progressive rise in antimicrobial resistance. Clarithromycin and metronidazole resistance now exceeds 30% worldwide, reaching over 60% and nearly 70%, respectively, in parts of Asia.^17,98^ Beyond treatment failure, broad-spectrum regimens disrupt the gut microbiota and may further drive resistance development.^99^ This scenario underscores the urgent need for alternative therapeutic strategies that target pathogen-specific vulnerabilities rather than targeting conserved bacterial functions.

Among these vulnerabilities, enzymatic pathways offer a vast yet underutilized reservoir of potential targets. While urease is the most extensively studied enzyme in *H. pylori* due to its essential role in acid resistance,^24,25^ the present review focused on nonurease pathways, aiming to map alternative enzymatic vulnerabilities that may support the development of new therapeutic strategies. Across the 49 included studies, we identified enzymes belonging to 21 metabolic or cellular pathways. Four of these pathways, purine metabolism, the shikimate pathway, the futalosine route, and nitrogen metabolism, accounted for more than half of all published studies, highlighting their prominence in current research. Notably, several enzymes within these pathways are essential for *H. pylori* survival, such as PNP and AdSS,^34^ as well as key enzymes of the futalosine pathway (e.g., MTAN), which represents the bacterium’s sole route for menaquinone biosynthesis.^55^ In contrast, other targets, although not strictly essential, significantly impair bacterial growth or virulence when inhibited and may therefore serve as adjunctive or combination targets, such as carbonic anhydrases^100^ and cytotoxin-associated pathogenicity island protein 1 (CagA),^101^ particularly in the context of multitarget inhibition strategies.

The predominance of these four pathways is not incidental but reflects a convergence of biological relevance and drug-development feasibility. This focus is driven by key features that have historically guided antimicrobial target selection, including essentiality for bacterial survival, divergence from human metabolism, and the availability of biochemical and structural data that support rational inhibitor design.^102,103^ In this regard, *H. pylori* is a purine auxotroph and depends entirely on salvage pathways, making enzymes such as PNP, AdSS, and XGHPRT indispensable for proliferation.^34^ Similarly, the shikimate and futalosine pathways, both absent in humans, are essential for aromatic amino acid and menaquinone biosynthesis, respectively, providing inherent selectivity for antimicrobial development.^48,56^ Finally, nitrogen metabolism also plays a critical role in acid acclimation and gastric colonization, further supporting its relevance as a therapeutic target.^63^

Beyond these individual features, the therapeutic relevance of these pathways is further reinforced by their metabolic interconnections, which amplify the impact of enzymatic inhibition. In the purine salvage network, PNP provides the purine bases that XGHPRT converts to inosine monophosphate (IMP), the central branching intermediate. IMP is subsequently converted into guanosine monophosphate (GMP) by IMPDH and into adenosine monophosphate (AMP) by AdSS.^34^ Because *H. pylori* lacks a *de novo* purine biosynthesis pathway, inhibition of any enzyme within this axis substantially reduces GMP and AMP production, thereby restricting the GTP and ATP pools required for DNA and RNA synthesis.^32,34^ This lack of metabolic redundancy increases pathway vulnerability and helps explain why these enzymes have been extensively explored as drug targets.

A second major point of metabolic interconnection involves the quinone biosynthesis. The shikimate pathway supplies chorismate, which is an indispensable precursor for initiating the futalosine pathway and assembling the aromatic core of menaquinone. Inhibiting shikimate enzymes therefore deprives the futalosine pathway of its entry substrate,^104,105^ effectively collapsing the first committed step of menaquinone synthesis. Complementing this, the methylerythritol phosphate (MEP) pathway generates the isoprenoid building blocks IPP and DMAPP, which are polymerized into the polyisoprene side chain subsequently attached to the futalosine-derived core.^106^ Thus, shikimate, futalosine, and MEP enzymes function at sequential but interdependent stages of the same biosynthetic process; inhibition of any of these pathways blocks menaquinone assembly from either the core or the side-chain direction. Because menaquinone is the central electron carrier of the *H. pylori* respiratory chain,^55^ multinode inhibition within this interconnected network has the potential to severely disrupt electron flux and cellular energy production.

Beyond these directly interconnected pathways, numerous other enzymes support complementary physiological functions essential for viability. Redox maintenance relies on both the menaquinone-dependent electron transport chain and the thioredoxin system.^79,80^ Peptidoglycan remodeling, DNA replication, and acid acclimation depend on enzymes such as glutamate racemase,^68,69^ DL-carboxypeptidase (Csd4),^83^ the β-sliding clamp,^78^ and carbonic anhydrases.^66–70^ Virulence factors such as HtrA and CagA further modulate host interaction and immune evasion.^82,84^ Although these pathways are not linearly connected, their functional complementarity suggests that coordinated inhibition across multiple targets may produce additive or synergistic effects, reinforcing the potential of multitarget therapeutic strategies.^107^

While such multitarget approaches are promising, ensuring selective inhibition relative to human homologues remains a central consideration in target prioritization. Among the 28 enzymes identified in this Review, 54% lacked human counterparts, representing inherently selective targets. Among those with human homologues, 62% were experimentally compared against both bacterial and human isoforms, and nearly all demonstrated substantially higher potency toward the *H. pylori* enzyme. For enzymes without direct selectivity assays, such as high-temperature requirement A serine protease (HtrA), PNP, AdSS, XGHPRT, and thioredoxin reductase (TrxR), structural differences described in the literature support the feasibility of selective inhibition. These include alternative isozyme configurations in AdSS,^108^ distinct oligomeric states in PNP,^37^ and unique catalytic architectures in TrxR^109^ and HtrA.^110^ Together, these findings indicate that most enzymes covered in this review either lack human homologues or exhibit sufficient divergence to allow species-specific inhibition.

Importantly, the presence of a human homologue does not preclude therapeutic viability.^111,112^ A well-established example is trimethoprim, a clinically used antimicrobial agent, which selectively inhibits bacterial dihydrofolate reductase (DHFR) despite the presence of a human ortholog, being approximately 50,000–100,000 times more active against the bacterial enzyme, a selectivity driven by subtle active-site differences.^111,113^ Although some off-target effects have been reported, trimethoprim is generally well tolerated, illustrating that selective inhibition of homologous targets can be achieved with an acceptable safety profile.^114^ Consistent with these observations, studies included in this review show that selective inhibition can be achieved for targets with human counterparts, as illustrated by representative examples including *H. pylori* α-carbonic anhydrase,^66–70^ dihydroorotate dehydrogenase,^76^ and α-1,3-fucosyltransferases.^88^

Moreover, differential metabolic dependency further contributes to selectivity.^103^ Many microorganisms, including *H. pylori*, rely on pathways that are either absent or nonessential in humans, whereas human cells often possess redundant metabolic routes that mitigate the effects of partial enzyme inhibition.^112^ This concept is exemplified by PNP inhibitors such as immucillins, which inhibit both human and microbial enzymes but display limited systemic toxicity due to differences in metabolic dependency and pathway redundancy between host and pathogen.^115,116^

In addition to these considerations, structural insights further support the identification and optimization of enzyme-targeting compounds. Analysis of structural and binding-related data across the included studies provides insight into the molecular features associated with effective enzyme inhibition (Table S3). Although the reported compounds target diverse enzymes, several recurring characteristics can be identified, including the presence of functional groups capable of forming hydrogen bonds with catalytic or substrate-binding residues, hydrophobic moieties that enhance interactions within enzyme pockets, and structural motifs that enable stabilization of ligand–enzyme complexes. These features highlight key pharmacophoric elements that may guide the rational design of new inhibitors and support the application of computational and *in silico* approaches for the identification of potent compounds across different target classes.

Despite these promising observations, the translational advancement of enzyme-targeting strategies for *H. pylori* remains limited. Cytotoxicity assessment was inconsistently reported across the included studies, and only three enzymes have been evaluated in infected animal models (Table S2). Accordingly, the available evidence, as summarized in Tables S1 and S2, should be interpreted as demonstrating the feasibility of selective enzymatic targeting rather than definitive proof of safety.

Among the enzymes tested *in vivo*, FDTS and DHODH inhibitors reduced bacterial load in mice, providing preliminary evidence of efficacy.^76,81^ The observation that these inhibitors lowered colonization levels is particularly relevant, as it offers functional *in vivo* validation of these pathways and demonstrates that enzymatic inhibition can translate into measurable effects within the gastric environment.^117^ These findings suggest that such compounds represent robust starting points for further optimization and are among the most promising therapeutic candidates currently available for *H. pylori*. Nonetheless, the limited number of *in vivo* studies reveals a substantial gap between mechanistic characterization and translational development. Bridging this gap will require coordinated efforts combining structural biology, medicinal chemistry, enzymatic and cell-based assays, *H. pylori* infection models, and comprehensive assessments of compound stability, permeability, and bioavailability.

In summary, the current landscape indicates that *H. pylori* relies on a limited set of nonredundant metabolic and virulence-associated pathways that remain underexploited. Targeting these pathways, especially those with essential functions, limited redundancy, and high divergence from human proteins, offers promising routes for rational antimicrobial development and may ultimately contribute to the identification of more selective and effective therapeutic candidates.

## 5 Conclusion

In light of these considerations, this systematic review provides an integrated assessment of nonurease enzymatic targets in *Helicobacter pylori*, highlighting several underexplored metabolic and structural pathways with relevant therapeutic potential. By consolidating evidence on essential pathways, metabolic interdependencies, and structural features that enable selective inhibition, this review offers guidance for the rational design and optimization of new inhibitors. Although preclinical evaluation remains limited, the findings compiled here support the identification of promising candidate targets and may inform future efforts aimed at developing mechanism-based therapeutic strategies capable of overcoming antimicrobial resistance and improving eradication outcomes.

## Supplementary Material



## Notes

Enzymatic inhibition parameters and antibacterial activity of selected *H. pylori* inhibitors (Table S1); extracted cytotoxicity and *in vivo* data for enzyme-targeted compounds (Table S2); structural and pharmacophoric features of nonurease targets in *Helicobacter pylori* (Table S3) (PDF)
