# Supplementary material for: Beyond Urease: New Potential Enzymatic Targets in Helicobacter pylori
Source: ACS Omega. 2026 Jun 4;11(23):33320–32. doi: 10.1021/acsomega.6c00763 (PMC13280913; doi:10.1021/acsomega.6c00763)
Supplement: Supplementary file 1 [file ao6c00763_si_001.pdf]

Supporting material

Beyond Urease: New Potential Enzymatic Targets in *Helicobacter pylori*

Ana Micaela Camini[1,2], Luiza Rosa Cogo[1], Maria Eduarda Delawi[1,2], Débora Bublitz Anton[2], Juliana Koakovski Acosta[1], Jeferson Camargo de Lima[1,2,3], Luís Fernando Saraiva Macedo Timmers[1,2,3*]

[1]Universidade do Vale do Taquari, Univates - Lajeado, Rio Grande do Sul, Brazil

[2]Programa de Pós-Graduação em Biotecnologia (PPGBiotec), Univates - Lajeado, Rio Grande do Sul, Brazil

[3]Programa de Pós-Graduação em Ciências Médicas (PPGCM), Univates - Lajeado, Rio Grande do Sul, Brazil

*Corresponding author: e-mail: luis.timmers@univates.br

Table S1. Enzymatic inhibition parameters and antibacterial activity of selected *H. pylori* inhibitors.

Table S2. Extracted cytotoxicity and *in vivo* data for enzyme-targeted compounds.

Table S3: Structural and pharmacophoric features of non-urease targets in *Helicobacter pylori.*

Table S1. Enzymatic inhibition parameters and antibacterial activity of selected *H. pylori* inhibitors.

Protein	Compound name / ID	Enzyme Inhibition				Bacterial Growth Inhibition				Reference
		Ki (µM)	Kd (µM)	Inhibition type	IC_{50} (µM)	*H. pylori* strain	MIC (µg/mL)	MBC (µg/mL)	IC_{50} (µM)	
Purine nucleoside phosphorylase (PNP)	2,6-diCl-Pu	22.2 ± 1.4	-	Non competitive	-	ATCC 26695	473	-	-	35
	6-BnS-Pu	7.9 ± 0.4	-		-		48	-	-	
	6BnO-2-Cl-Pu	Kic: 18.3 ± 7.3<br>Kiu = 4.6 ± 0.5	-	Kic = Competitive<br>Kiu = Non competitive	-		-	-	-	
	6BnS-2Cl-Pu-9dr	Kic: 6.2 ± 2.4<br>Kiu: 2.9 ± 0.5	-	Kic = Competitive<br>Kiu = Non competitive	-		79	-	-	
	6BnS-2Cl-Pu	Kiu = 1.8 ± 0.2	-	Non competitive	-		69	-	-	
Purine nucleoside phosphorylase (PNP)	Formycin A	14.0 ± 1.7	-	Competitive	-	-	-	-	-	36
Purine nucleoside phosphorylase (PNP)	Formycin B	0.96 ± 0.08	-	-	-	ATCC 26695	>1000	-	-	37
						SS1 strain	≥ 397			
	Formycin A	14.0 ± 1.7	-	-	-	ATCC 26695	≥ 870	-	-	
						SS1 strain	> 1000			
Adenylosuccinate synthetase (AdSS)	Hadacidin	0.19 ± 0.02	-	-	-	ATCC 26695	>1000	-	-	
						SS1 strain	>1000			
	FA/FB 1:1	-	-	-	-	ATCC 26695	>500/500	-	-	

Protein	Compound name / ID	Enzyme Inhibition				Bacterial Growth Inhibition				Reference
		Ki (μM)	Kd (μM)	Inhibition type	IC_{50} (μM)	*H. pylori* strain	MIC (μg/mL)	MBC (μg/mL)	IC_{50} (μM)	
						SS1 strain	>177/177			
	FA/Hada 1:1	-	-	-	-	ATCC 26695	>500/500	-	-	
						SS1 strain	>500/500			
	FB/Hada 1:1	-	-	-	-	ATCC 26695	>500/500	-	-	
						SS1 strain	>500/500			
Adenylosuccinate synthetase (AdSS)	Hadacidin	0.19 ± 0.002	-	Competitive	-	-	-	-	-	38
Adenylosuccinate synthetase (AdSS)	Pyridoxal 5'-phosphate	6.95 ± 0.82	-	Competitive	9.97	ATCC 26695	370	740	-	39
						N6	370	370		
						P12	370	370		
						M91 (clarithromycin and metronidazole resistance)	≥309	≤370		
						M92 (clarithromycin resistance)	≥185	≤370		
						M93 (metronidazole resistance)	185	185		
						M26 (clarithromycin and	≥309	185		

Protein	Compound name / ID	Enzyme Inhibition				Bacterial Growth Inhibition				Reference
		Ki (μM)	Kd (μM)	Inhibition type	IC_{50} (μM)	*H. pylori* strain	MIC (μg/mL)	MBC (μg/mL)	IC_{50} (μM)	
						metronidazole resistance)				
Xanthine-guanine phosphoribosyltransferase (XGHPRT)*	I	0.2 ± 0.05	-	-	-	ATCC 26695	-	-	-	40
	III	0.3 ± 0.04	-	-	-		-	-	-	
	VI	1.9 ± 0.4	-	-	-		-	-	-	
	VIII	3.4 ± 0.4	-	-	-		-	-	-	
	X	5 ± 1	-	-	-		-	-	-	
Inosine-5'-monophosphate dehydrogenase (IMPDH)	Irigenin	-	-	-	2.07 ± 1.90	ATCC 700392	3.9	-	-	41
	Orientin	-	-	-	-		15.53	-	-	
	Tectorigenin	-	-	-	-		15.63	-	-	
	Tectoridin	-	-	-	-		>125	-	-	
Inosine-5'-monophosphate dehydrogenase (IMPDH)	6	-	-	-	2.42	ATCC 26695	1.95	-	-	42
	10	-	-	-	4.48		3.9	-	-	
	11	-	-	-	2.56		7.81	-	-	
	21	-	-	-	2.95		7.81	-	-	
	20	-	-	-	-		15.63	-	-	
Inosine-5'-monophosphate dehydrogenase (IMPDH)	10	-	-	-	8.9 ± 0.03	-	-	-	-	43
	24	-	-	-	2.21 ± 0.02	-	-	-	-	
	(±)-47	-	-	-	10.7 ± 0.12	-	-	-	-	
	(±)-48	-	-	-	7.2 ± 0.07	-	-	-	-	
	(±)-49	-	-	-	8.6 ± 0.03	-	-	-	-	
Inosine-5'-monophosphate	1	-	-	-	0.8 ± 0.02	-	-	-	-	44
	2	-	-	-	1 ± 0.03	-	-	-	-	

Protein	Compound name / ID	Enzyme Inhibition				Bacterial Growth Inhibition				Reference
		Ki (μM)	Kd (μM)	Inhibition type	IC_{50} (μM)	*H. pylori* strain	MIC (μg/mL)	MBC (μg/mL)	IC_{50} (μM)	
dehydrogenase (IMPDH)	3	-	-	-	1.60 ± 0.03	-	-	-	-	
	4	-	-	-	3.35 ± 0.04	-	-	-	-	
	5	-	-	-	4.38 ± 0.03	-	-	-	-	
Inosine-5'-mono phosphate dehydrogenase (IMPDH)	1	-	-	Non competitive	0.155 ± 0.082	-	-	-	-	45
	2	-	-	Non competitive	1.885 ± 0.42	-	-	-	-	
Inosine-5'-mono phosphate dehydrogenase (IMPDH)	PE-G-3	-	-	-	0.65	ATCC 26695	0.98	-	-	46
	PE-G-1	-	-	-	-		1.95	-	-	
	PE-G-2	-	-	-	-		15.63	-	-	
	PE-3	-	-	-	-		15.63	-	-	
	PE-1	-	-	-	-		62.5	-	-	
Inosine-5'-mono phosphate dehydrogenase (IMPDH)	7j	-	-	Non competitive	0.095 ±0.023	-	-	-	-	47
	7r	-	-	Non competitive	0.969 ±0.086	-	-	-	-	
	7i	-	-	Non competitive	2.067 ±0.551	-	-	-	-	
	7n	-	-	-	2.559 ±0.061	-	-	-	-	
	7d	-	-	-	2.674 ±0.0066	-	-	-	-	
Shikimate kinase (SK)	3e - 10e (prodrug)	0.46 ± 0.02	-	Competitive	-	Clinical isolate from Guadalajara Hospital	128	-	-	48

Protein	Compound name / ID	Enzyme Inhibition				Bacterial Growth Inhibition				Reference
		Ki (µM)	Kd (µM)	Inhibition type	IC_{50} (µM)	*H. pylori* strain	MIC (µg/mL)	MBC (µg/mL)	IC_{50} (µM)	
	3i - 10i (prodrug)	0.56 ± 0.05	-	Competitive	-	Clinical isolate from Guadalajara Hospital	4	-	-	
	3b	1.00 ± 0.05	-	Competitive	-	-	-	-	-	
	3c	1.28 ± 0.03	-	Competitive	-	-	-	-	-	
	3h	1.80 ± 0.10	-	Competitive	-	-	-	-	-	
Shikimate kinase (SK)	4b	15.5 ± 1.1	-	Competitive	-	-	-	-	-	50
	4c	9.2 ± 1.0	-	Competitive	-	-	-	-	-	
	4d	12 ± 2	-	Competitive	-	-	-	-	-	
	4e	10 ± 0.6	-	Competitive	-	-	-	-	-	
	4f	5.0 ± 0.3	-	Competitive	-	-	-	-	-	
Shikimate kinase (SK)	4b	0.30 ± 0.02	-	-	-	-	-	-	-	51
	3a	0.56 ± 0.05	-	-	-	-	-	-	-	
	3c	1.80 ± 0.10	-	-	-	-	-	-	-	
	4a	5.20 ± 0.40	-	-	-	-	-	-	-	
	4d	8.70 ± 0.50	-	-	-	-	-	-	-	

Protein	Compound name / ID	Enzyme Inhibition				Bacterial Growth Inhibition				Reference
		Ki (μM)	Kd (μM)	Inhibition type	IC_{50} (μM)	*H. pylori* strain	MIC (μg/mL)	MBC (μg/mL)	IC_{50} (μM)	
Shikimate kinase (SK)	Rosmarinicacid	-	-	-	-		MIC90: 800	-	-	
Urease	Oroxindin	-	-	-	-	ATCC 43504	MIC90: 50	-	-	52
Aspartate-semi aldehyde dehydrogenase	Verbascoside	-	-	-	-		MIC90: 1200	-	-	
	ZINC03848012	-	-	-	-	ATCC 43504	28 ± 5	-	-	
						ATCC 700392	21 ± 7			
	ZINC00608186	-	-	-	-	ATCC 43504	8 ± 2	-	-	
						ATCC 700392	15 ± 4			
Shikimate dehydrogenase (SDH)	ZINC00119988	-	-	-	-	ATCC 43504	36 ± 7	-	-	53
						ATCC 700392	29 ± 5			
	ZINC14512219	-	-	-	-	ATCC 43504	45 ± 6	-	-	
						ATCC 700392	30 ± 4			
	ZINC00118133	-	-	-	-	ATCC 43504	37 ± 6	-	-	
						ATCC 700392	49 ± 8			
	15	19 ± 2	-	-	-	-	-	-	-	
	14	85 ± 5	-	-	-	-	-	-	-	
3-dehydroquinase dehydratase type II (DHQase II)	17r	52 ± 5	-	-	-	-	-	-	-	54
	18	18 ± 2	-	-	-	-	-	-	-	
	19	6.3 ± 0.2	-	-	-	-	-	-	-	
	15	-	0.00005 ± 0.00002	-	-	-	-	-	-	
Aminodeoxyfutalosine										57

Protein	Compound name / ID	Enzyme Inhibition				Bacterial Growth Inhibition				Reference
		Ki (µM)	Kd (µM)	Inhibition type	IC_{50} (µM)	*H. pylori* strain	MIC (µg/mL)	MBC (µg/mL)	IC_{50} (µM)	
nucleosidase (MTAN)	16	-	0.00002 ± 0.000006	-	-	-	-	-	-	
	30	-	0.00003 ± 0.00001	-	-	-	-	-	-	
	32	-	0.00004 ± 0.00001	-	-	-	-	-	-	
	26	-	0.00002 ± 0.000002	-	-	-	-	-	-	
Aminodeoxyfutalosine nucleosidase (MTAN)	HT-DADMe-ImmA (14)	-	-	-	-	ATCC 700824	-	-	0.035 ± 0.005	58
Aminodeoxyfutalosine nucleosidase (MTAN)	2	0.00019 ± 0.00007	-	-	-	-	-	-	IC90 = 6-12 ng/mL	59
	22	0.00034 ± 0.00007	-	-	-	-	-	-	IC90 = 9 ng/mL	
	32	0.000043 ± 0.000001	-	-	-	-	-	-	IC90 = 8 ng/mL	
	36	-	-	-	-	-	-	-	-	

Protein	Compound name / ID	Enzyme Inhibition				Bacterial Growth Inhibition				Reference
		Ki (µM)	Kd (µM)	Inhibition type	IC_{50} (µM)	*H. pylori* strain	MIC (µg/mL)	MBC (µg/mL)	IC_{50} (µM)	
	54	0.000030 ± 0.000003	-	-	-	-	-	-	IC_{90} = 8 ng/mL	
Aminodeoxyfutalosine nucleosidase (MTAN)	2	0.00009	-	-	-	-	-	-	-	60
	6	0.00028	-	-	-	-	-	-	-	
	5	0.00031	-	-	-	-	-	-	-	
	4	0.00074	-	-	-	-	-	-	-	
	3	0.00079	-	-	-	-	-	-	-	
Aminofutalosine deaminase (AFLDA)	(8R)-CF	-	-	-	-	ATCC 700824	-	-	-	61
	(8R)-MTCF	0.063 ± 0.004	-	-	-	ATCC 700825	-	-	14 ± 1	
	(8R)-MT-2′d-CF	-	-	-	-	ATCC 700826	-	-	-	
	(8R)-PrT-2′d-CF	0.42 ± 0.14	-	-	-	ATCC 700827	-	-	-	
	(8R)-PhT-2′d-CF	0.85 ± 0.2	-	-	-	ATCC 700828	-	-	-	
Aminodeoxyfutalosine/aminofutalosine synthase (MqnE)	12	-	-	-	16.1 ± 3.9	-	-	-	16.1 ± 3.9	62
	9	3.1 ± 0.1	-	-	1.8 ± 0.4	-	-	-	1.8 ± 0.4	
Carbonic anhydrases α and β (αCA and βCA)	Carvacrol	HpCAα = 8.4 HpCAβ = 13.3	-	Competitive	-	ATCC 43504	128	256	-	66

Protein	Compound name / ID	Enzyme Inhibition				Bacterial Growth Inhibition				Reference
		Ki (µM)	Kd (µM)	Inhibition type	IC_{50} (µM)	*H. pylori* strain	MIC (µg/mL)	MBC (µg/mL)	IC_{50} (µM)	
	Thymol	HpCAα > 100 HpCAβ = 3.4	-	Competitive	-		128	256	-	
Carbonic anhydrase α (αCA)	4c	0.79	-	-	-	-	-	-	-	67
	5c	1.63	-	-	-	-	-	-	-	
	5b	1.64	-	-	-	-	-	-	-	
	4f	1.73	-	-	-	-	-	-	-	
	4a	1.95	-	-	-	-	-	-	-	
Carbonic anhydrase α (αCA)	3	0.82	-	-	-	-	-	-	-	68
	1	0.323	-	-	-	-	-	-	-	
	2	0.549	-	-	-	-	-	-	-	
Carbonic anhydrase α (αCA)	Acetazolamide	0.021	-	-	-	-	-	-	-	69
	Methazolamide	0.225	-	-	-	-	-	-	-	
Carbonic anhydrase α (αCA)	17a	0.052	-	-	-	ATCC 43504	>128	>128	-	70
	17b	0.055	-	-	-	ATCC 43504	>128	>128	-	
	11b	0.057	-	-	-	ATCC 43504	>128	>128	-	
	19b	0.203	-	-	-	ATCC 43504	8	32	-	
						F1 Clinical isolate	16	32		
	20a	0.093	-	-	-	ATCC 43504	16	32	-	
						F1 Clinical isolate	16	32		

Protein	Compound name / ID	Enzyme Inhibition				Bacterial Growth Inhibition				Reference
		Ki (μM)	Kd (μM)	Inhibition type	IC_{50} (μM)	*H. pylori* strain	MIC (μg/mL)	MBC (μg/mL)	IC_{50} (μM)	
Glutamate racemase	Pyridodiazepine amine inhibitor	-	-	-	-	Clinical isolate	0.13 to 0.5	-	-	71
						SS1	0.25			
						ARHp80 (clinical isolate)	0.25			
						Isogenic MurI-overexpressing strain of ARHp80	64			
						Isogenic efflux compromised strain of ARHp80	0.13			
Glutamate racemase	NP-004604	-	170 ± 2	-	425.3	-	-	-	-	72
	NP-020560	-	54.7 ± 0.3	-	6.6 ± 3.1	-	-	-	-	
	NP-000205	-	80.1 ± 0.6	-	512.8	-	-	-	-	
	NP-004431	-	228 ± 2	-	705.3	-	-	-	-	
	NP-008029	-	910 ± 10	-	-	-	-	-	-	
NADH-quinone oxidoreductase subunit D	25	-	-	-	-	ATCC 43504	-	-	7.7 ± 0.070 M	73
		-	-	-	-	SS1	-	-	7.7 ± 0.044 M	
		-	-	-	-	SS1 NuoDA402P	-	-	6,9 ± 0.15 M	
	26	-	-	-	-	ATCC 43504	-	-	8.0 ± 0.13 M	

Protein	Compound name / ID	Enzyme Inhibition				Bacterial Growth Inhibition				Reference
		Ki (μM)	Kd (μM)	Inhibition type	IC_{50} (μM)	*H. pylori* strain	MIC (μg/mL)	MBC (μg/mL)	IC_{50} (μM)	
		-	-	-	-	SS1	-	-	8.2 ± 0.093 M	
		-	-	-	-	SS1 NuoDA402P	-	-	7.1 ± 0.20 M	
Bifunctional enzyme IspD/IspF	4	-	-	-	13 ± 2	P12	12.5	-	3.3 ± 0.9	74
	5	-	-	-	3.7 ± 0.7		12.5	-	5.8 ± 1.7	
	6	-	-	-	5.3 ± 1.0		25	-	7.2 ± 1.6	
	7	-	-	-	3.8 ± 0.4		50	-	12 ± 4	
	8	-	-	-	15 ± 3		50	-	69 ± 18	
Bifunctional enzyme IspD/IspF	Rosmarinic acid	Substrate CTP= 2.26 ± 0.16 Substrate MEP= 0.49 ± 0.06	-	Substrate CTP= non competitive Substrate MEP= competitive	2.74 ± 0.17	-	-	-	-	75
	Compound 1	-	-	-	3.79 ±0.30	-	-	-	-	
	Tanshinone IIA sulfonic sodium	Substrate CTP= 2.81 ± 0.33 Substrate MEP= 6.89 ± 0.96	-	Substrate CTP= non competitive Substrate MEP= competitive	17.80 ± 1.09	-	17.30 ± 2.43	-	-	

Protein	Compound name / ID	Enzyme Inhibition				Bacterial Growth Inhibition				Reference
		Ki (µM)	Kd (µM)	Inhibition type	IC_{50} (µM)	*H. pylori* strain	MIC (µg/mL)	MBC (µg/mL)	IC_{50} (µM)	
Dihydroorotate dehydrogenase (DHODH)	Intervenolin	-	-	-	11.11 ± 2.44	JCM 12093	0.0156	-	-	76
						JCM 12095	0.0156			
						SS1	0.125			
						ATCC 49503	0.125			
						ATCC 700684	0.125			
						ATCC 43504	1			
	AS-1664	-	-	-	0.06 ± 0.02	JCM 12093	0.0312	-	-	
						JCM 12095	0.0312			
						SS1	0.0312			
						ATCC 49503	0.0312			
						ATCC 700684	0.0156			
						ATCC 43504	0.0625			
	AS1934	-	-	-	4.55	JCM 12093	0.125	-	-	
						JCM 12095	0.25			
						SS1	0.125			
						ATCC 49503	0.125			
						ATCC 700684	0.125			
						ATCC 43504	0.5			
Pseudaminic acid metabolism (Pse)	CD23703	-	-	-	72	-	-	-	-	77
	CD09463	-	-	-	21.4	-	-	-	-	
	CD36508	-	-	-	15.1	-	-	-	-	
	CD26389	-	-	-	14.1	-	-	-	-	

Protein	Compound name / ID	Enzyme Inhibition				Bacterial Growth Inhibition				Reference
		Ki (μM)	Kd (μM)	Inhibition type	IC_{50} (μM)	*H. pylori* strain	MIC (μg/mL)	MBC (μg/mL)	IC_{50} (μM)	
	CD24868	-	-	-	12.3	-	-	-	-	
β Sliding Clamp	5-chloroisatin	-	-	Competitive	-	ATCC 26695	18 μM	-	-	78
	3,4-difluorobenzamide	-	-	Competitive	-		824 μM	-	-	
Thioredoxin reductase (TrxR)	2	-	-	-	< 0,005	G27	0.30 - 0.65 μM	-	-	79
	3	-	-	-	< 0,005		0.30 - 0.65 μM	-	-	
	4	-	-	-	< 0,005		0.30 - 0.65 μM	-	-	
	5	-	-	-	Between 0.01 and 0.015		0.30 - 0.65 μM	-	-	
	6	-	-	-	< 0,005		5 μM	-	-	
Thioredoxin reductase (TrxR)	3c	-	-		-	G27	4.5 - 1.9	-	-	80
	4a	-	-		-		2.0 - 0	2.0 - 0	-	
	4c	-	-		-		2.0 - 0	2.0 - 0	-	
	5a	-	-		-		2.0 - 0	2.0 - 0	-	
	5c	-	-		-		2.0 - 0	2.0 - 0	-	
Flavin-dependent thymidylate synthase (FDTS)	007-A (C8-C1)	367	-	-	-	ATCC 26695	33.98 μM	-	-	81
						mouse-adapted strain SS1	33.98 μM			
	010-C	28	-	-	-	ATCC 26695	39.32 μM	-	-	
						mouse-adapted strain SS1	39.32 μM			
	010-E	1	-	-	-	ATCC 26695	14.27 μM	-	-	
						mouse-adapted strain SS1	14.27 μM			

Protein	Compound name / ID	Enzyme Inhibition				Bacterial Growth Inhibition				Reference
		Ki (μM)	Kd (μM)	Inhibition type	IC_{50} (μM)	*H. pylori* strain	MIC (μg/mL)	MBC (μg/mL)	IC_{50} (μM)	
	010-I	258	-	-	-	ATCC 26695<br>mouse-adapted strain SS1	43.42 μM<br>43.42 μM	-	-	
High-temperature requirement A Serine protease (HtrA)	1a	-	27 ± 4	-	-	-	-	-	-	82
	1b	-	12	-	-	-	-	-	-	
	1c	-	10	-	-	-	-	-	-	
	2	-	37 ± 4	-	-	-	-	-	-	
	5		13	-	-	-	-	-	-	
DL-carboxypeptidase (csd4)	Phosphinic acid 1	1.5 ± 0.3	-	Competitive	-	-	-	-	-	83
Cytotoxin-associated pathogenicity island protein 1 (CagA)	1G2	-	-	Non competitive	196.2 (±0.026)	ATCC 26695**	-	-	-	84
	1G2#1	-	-	-	547.4 (±0.59)		-	-	-	
	1G2#2	-	-	-	619.6 (±0.95)		-	-	-	
	1G2#3	-	-	-	479.6 (±0.46)		-	-	-	
	1G2#4	-	-	Non competitive	81.9 (±0.6)		-	-	-	
γ-Glutamyltranspeptidase (gGT)	AHPP-butyric acid (2-Amino-4[methyl(4-trifluoromethylphenyl) phospono]-butyric acid)	0.013	-	-	-	-	-	-	-	85

Protein	Compound name / ID	Enzyme Inhibition				Bacterial Growth Inhibition				Reference
		Ki (µM)	Kd (µM)	Inhibition type	IC_{50} (µM)	*H. pylori* strain	MIC (µg/mL)	MBC (µg/mL)	IC_{50} (µM)	
	AMOP-butyric acid (2-Amino-4-[methyl(4-methylumbelliferyl) phosphono]butyric acid)	0.077	-	-	-	-	-	-	-	
	GGsTopTM	0.201	-	-	-	-	-	-	-	
	DL-AP4-butyric acid (DL-2-Amino-4-phosphonobutyric acid)	0.950	-	-	-	-	-	-	-	
	Quisqualic acid	115	-	-	-	-	-	-	-	
Glucose-6-phosphate 1-dehydrogenase (G6PD)	YGC-1 scaffold 1,3-thiazolidine-2,4-dione	Inhibition (%) at (400 µM) = 84	-	Non competitive	310	-	-	-	-	86
	MDG-1 1H-benzimidazole	Inhibition (%) at (400 µM) = 84	-	Non competitive	465	-	-	-	-	
	MGD-2 1,3-benzoxazole	Inhibition (%) at (400 µM) = 84	-	Non competitive	340	-	-	-	-	

Protein	Compound name / ID	Enzyme Inhibition				Bacterial Growth Inhibition				Reference
		Ki (μM)	Kd (μM)	Inhibition type	IC_{50} (μM)	*H. pylori* strain	MIC (μg/mL)	MBC (μg/mL)	IC_{50} (μM)	
	TDA-1 morpholine	Inhibition (%) at (400 μM) = 84	-	Non competitive	204	-	-	-	-	
	JMM3 biphenylcarbonitrile	Inhibition (%) at (400 μM) = 84	-	Non competitive	304	-	-	-	-	
Methionine/methionyl aminopeptidase (metAP)	7d	0.53 ± 0.073	-	-	-	-	-	-	-	87
	7 e5	0,62 ± 0.096	-	-	-	-	-	-	-	
	7d4	0.11 ± 0.010	-	-	-	-	-	-	-	
	7g7	0.14 ± 0.050	-	-	-	-	-	-	-	
	7e	0.07 ± 0.024	-	-	-	-	-	-	-	
α-1,3-fucosyltransferase	3	> 45% between 0 - 400	-	-	-	-	-	-	-	88
Pyruvate ferredoxin oxidoreductase (PFOR)	Amixicile	Inhibition 40 μM: 75%	-	-	-	ATCC 26695	0.5	-	-	89
	4.6	Inhibition 40 μM: 95%	-	-	-		0.5	-	-	

Protein	Compound name / ID	Enzyme Inhibition				Bacterial Growth Inhibition				Reference
		Ki (μM)	Kd (μM)	Inhibition type	IC_{50} (μM)	*H. pylori* strain	MIC (μg/mL)	MBC (μg/mL)	IC_{50} (μM)	
	4.8	Inhibition 40 μM: 93%	-	-	-		125	-	-	
	4.13	Inhibition 40 μM: 75%	-	-	-		0.5	-	-	
	4.16	Inhibition 40 μM: 94%	-	-	-		0.5	-	-	

Notes:
*All compounds presented in the table inhibited bacterial growth at a concentration of 50 μM.
**Have no negative effect on the growth of *H. pylori* on solid agar media at concentrations up to 500 μM
Abbreviations:
Ki: Inhibition constant (Kic: Competitive inhibition constant; Kiu: Uncompetitive inhibition constant)
Kd: Dissociation constant
IC_{50} : Half maximal inhibitory concentration
MIC: Minimum inhibitory concentration
MBC: Minimum bactericidal concentration
LC_{50} : Median lethal concentration
TC_{50} : Toxic concentration 50
SS1 strain: Sydney Strain

Table S2. Extracted cytotoxicity and *in vivo* data for enzyme-targeted compounds

Protein	Compound name / ID	Cytotoxicity assays				*In vivo* assays		Reference
		Cell line	LC_{50} (μM)	TC_{50}	IC_{50} (μM)	Animal model	Effect on Colonization	
Purine nucleoside phosphorylase (PNP)	2,6-diCl-Pu	-	-	-	-	-	-	35
	6-BnS-Pu	-	-	-	-	-	-	
	6BnO-2-Cl-Pu	-	-	-	-	-	-	
	6BnS-2Cl-Pu-9dr	-	-	-	-	-	-	
	6BnS-2Cl-Pu	-	-	-	-	-	-	
Purine nucleoside phosphorylase (PNP)	Formycin A	-	-	-	-	-	-	36
Purine nucleoside phosphorylase (PNP)	Formycin B	-	-	-	-	-	-	37
	Formycin A	-	-	-	-	-	-	
Adenylosuccinate synthetase (AdSS)	Hadacidin	-	-	-	-	-	-	
	FA/FB 1:1	-	-	-	-	-	-	
	FA/Hada 1:1	-	-	-	-	-	-	

Protein	Compound name / ID	Cytotoxicity assays				*In vivo* assays		Reference
		Cell line	LC_{50} (μM)	TC_{50}	IC_{50} (μM)	Animal model	Effect on Colonization	
	FB/Hada 1:1	-	-	-	-	-	-	
Adenylosuccinate synthetase (AdSS)	Hadacidin	-	-	-	-	-	-	38
Adenylosuccinate synthetase (AdSS)	Pyridoxal 5'-phosphate	-	-	-	-	-	-	39
Xanthine-guanine phosphoribosyltransferase (XGHPRT)*	I	-	-	-	-	-	-	40
	III	-	-	-	-	-	-	
	VI	-	-	-	-	-	-	
	VIII	-	-	-	-	-	-	
	X	-	-	-	-	-	-	
Inosine-5'-monophosphate dehydrogenase (IMPDH)	Irigenin	-	-	-	-	-	-	41
	Orientin	-	-	-	-	-	-	
	Tectorigenin	-	-	-	-	-	-	
	Tectoridin	-	-	-	-	-	-	

Protein	Compound name / ID	Cytotoxicity assays				*In vivo* assays		Reference
		Cell line	LC_{50} (μM)	TC_{50}	IC_{50} (μM)	Animal model	Effect on Colonization	
Inosine-5'-monophosphate dehydrogenase (IMPDH)	6	-	-	-	-	-	-	42
	10	-	-	-	-	-	-	
	11	-	-	-	-	-	-	
	21	-	-	-	-	-	-	
	20	-	-	-	-	-	-	
Inosine-5'-monophosphate dehydrogenase (IMPDH)	10	-	-	-	-	-	-	43
	24	-	-	-	-	-	-	
	(±)-47	-	-	-	-	-	-	
	(±)-48	-	-	-	-	-	-	
	(±)-49	-	-	-	-	-	-	
Inosine-5'-monophosphate dehydrogenase (IMPDH)	1	-	-	-	-	-	-	44
	2	-	-	-	-	-	-	
	3	-	-	-	-	-	-	
	4	-	-	-	-	-	-	
	5	-	-	-	-	-	-	
Inosine-5'-monophosphate	1	-	-	-	-	-	-	45
	2	-	-	-	-	-	-	

Protein	Compound name / ID	Cytotoxicity assays				*In vivo* assays		Reference
		Cell line	LC_{50} (μM)	TC_{50}	IC_{50} (μM)	Animal model	Effect on Colonization	
dehydrogenase (IMPDH)								
Inosine-5'-monophosphate dehydrogenase (IMPDH)	PE-G-3	-	-	-	-	-	-	46
	PE-G-1	-	-	-	-	-	-	
	PE-G-2	-	-	-	-	-	-	
	PE-3	-	-	-	-	-	-	
	PE-1	-	-	-	-	-	-	
Inosine-5'-monophosphate dehydrogenase (IMPDH)	7j	Cytotoxicity HEK29 (Tested at a concentration of 10 μM)	None	-	-	-	-	47
	7r		None	-	-	-	-	
	7i		None	-	-	-	-	
	7n		None	-	-	-	-	
	7d		None	-	-	-	-	
Shikimate kinase (SK)	3e - 10e (prodrug)	-	-	-	-	-	-	48
	3i - 10i (prodrug)	-	-	-	-	-	-	
	3b	-	-	-	-	-	-	
	3c	-	-	-	-	-	-	
	3h	-	-	-	-	-	-	

Protein	Compound name / ID	Cytotoxicity assays				*In vivo* assays		Reference
		Cell line	LC_{50} (μM)	TC_{50}	IC_{50} (μM)	Animal model	Effect on Colonization	
Shikimate kinase (SK)	4b	-	-	-	-	-	-	50
	4c	-	-	-	-	-	-	
	4d	-	-	-	-	-	-	
	4e	-	-	-	-	-	-	
	4f	-	-	-	-	-	-	
Shikimate kinase (SK)	4b	-	-	-	-	-	-	51
	3a	-	-	-	-	-	-	
	3c	-	-	-	-	-	-	
	4a	-	-	-	-	-	-	
	4d	-	-	-	-	-	-	
Shikimate kinase (SK)	Rosmarinic acid	-	-	-	-	-	-	52
Urease	Oroxindin	-	-	-	-	-	-	
Aspartate-semialdehyde dehydrogenase	Verbascoside	-	-	-	-	-	-	
Shikimate dehydrogenase (SDH)	ZINC03848012	-	-	-	-	-	-	53

Protein	Compound name / ID	Cytotoxicity assays				*In vivo* assays		Reference
		Cell line	LC_{50} (μM)	TC_{50}	IC_{50} (μM)	Animal model	Effect on Colonization	
	ZINC00608186	-	-	-	-	-	-	
	ZINC00119988	-	-	-	-	-	-	
	ZINC14512219	-	-	-	-	-	-	
	ZINC00118133	-	-	-	-	-	-	
3-dehydroquinase dehydratase type II (DHQase II)	15	-	-	-	-	-	-	54
	14	-	-	-	-	-	-	
	17r	-	-	-	-	-	-	
	18	-	-	-	-	-	-	
	19	-	-	-	-	-	-	
Aminodeoxyfutalosine nucleosidase (MTAN)	15	-	-	-	-	-	-	57
	16	-	-	-	-	-	-	
	30	-	-	-	-	-	-	
	32	-	-	-	-	-	-	
	26	-	-	-	-	-	-	

Protein	Compound name / ID	Cytotoxicity assays				*In vivo* assays		Reference
		Cell line	LC_{50} (μM)	TC_{50}	IC_{50} (μM)	Animal model	Effect on Colonization	
Aminodeoxyfutalosine nucleosidase (MTAN)	HT-DADMe-ImmA (14)	-	-	-	-	-	-	58
Aminodeoxyfutalosine nucleosidase (MTAN)	2	-	-	-	-	-	-	59
	22	-	-	-	-	-	-	
	32	-	-	-	-	-	-	
	36	-	-	-	-	-	-	
	54	-	-	-	-	-	-	
Aminodeoxyfutalosine nucleosidase (MTAN)	2	-	-	-		-	-	60
	6	-	-	-		-	-	
	5	-	-	-		-	-	
	4	-	-	-		-	-	
	3	-	-	-		-	-	
Aminofutalosine deaminase (AFLDA)	(8R)-CF	-	-	-	-	-	-	61
	(8R)-MTCF	-	-	-	-	-	-	
	(8R)-MT-2′d-CF	-	-	-	-	-	-	
	(8R)-PrT-2′d-CF	-	-	-	-	-	-	
	(8R)-PhT-2′d-CF	-	-	-	-	-	-	

Protein	Compound name / ID	Cytotoxicity assays				*In vivo* assays		Reference
		Cell line	LC_{50} (μM)	TC_{50}	IC_{50} (μM)	Animal model	Effect on Colonization	
Aminodeoxyfutalosine/aminofutalosine synthase (MqnE)	12	-	-	-	-	-	-	62
	9	-	-	-	-	-	-	
Carbonic anhydrases α and β (αCA and βCA)	Carvacrol	-	-	-	-	-	-	66
	Thymol	-	-	-	-	-	-	
Carbonic anhydrase α (αCA)	4c	-	-	-	-	-	-	67
	5c	-	-	-	-	-	-	
	5b	-	-	-	-	-	-	
	4f	-	-	-	-	-	-	
	4a	-	-	-	-	-	-	
Carbonic anhydrase α (αCA)	3	-	-	-	-	-	-	68
	1	-	-	-	-	-	-	
	2	-	-	-	-	-	-	
Carbonic anhydrase α (αCA)	Acetazolamide	-	-	-	-	-	-	69
	Methazolamide	-	-	-	-	-	-	
Carbonic anhydrase α (αCA)	17a	-	-	-	-	-	-	70
	17b	-	-	-	-	-	-	

Protein	Compound name / ID	Cytotoxicity assays				*In vivo* assays		Reference
		Cell line	LC_{50} (μM)	TC_{50}	IC_{50} (μM)	Animal model	Effect on Colonization	
	11b	-	-	-	-	-	-	
	19b	-	-	-	-	-	-	
	20a	-	-	-	-	-	-	
Glutamate racemase	Pyridodiazepine amine inhibitor	-	-	-	-	Mice	No decrease in *H. pylori* colonization was observed.	71
Glutamate racemase	NP-004604	-	-	-	-	-	-	72
	NP-020560	-	-	-	-	-	-	
	NP-000205	-	-	-	-	-	-	
	NP-004431	-	-	-	-	-	-	
	NP-008029	-	-	-	-	-	-	
NADH-quinone oxidoreductase subunit D	25	FaDu cells cytotoxicity pIC50 (M)	- - -	- - -	3.4 ± 0.050	- - -	- - -	73

Protein	Compound name / ID	Cytotoxicity assays				In vivo assays		Reference
		Cell line	LC_{50} (μM)	TC_{50}	IC_{50} (μM)	Animal model	Effect on Colonization	
			-	-		-	-	
	26		-	-	4.8 ± 0.00023	-	-	
			-	-		-	-	
Bifunctional enzyme IspD/IspF	4	Cell viability (30 uM) - Murine fibroblast	Cell viability (30uM) 46 ± 14	-	-	-	-	74
	5		71 ± 26	-	-	-	-	
	6		107 ± 5	-	-	-	-	
	7		30 ± 18	-	-	-	-	
	8		6.1 ± 1.7	-	-	-	-	
Bifunctional enzyme IspD/IspF	Rosmarinic acid	-	-	-	-	-	-	75
	Compound 1	-	-	-	-	-	-	
	Tanshinone IIA sulfonic sodium	-	-	-	-	-	-	
Dihydroorotate dehydrogenase (DHODH)	Intervenolin	-	-	-	-	-	-	76

Protein	Compound name / ID	Cytotoxicity assays				*In vivo* assays		Reference
		Cell line	LC_{50} (μM)	TC_{50}	IC_{50} (μM)	Animal model	Effect on Colonization	
	AS-1664	-	-	-	-	-	Exhibited a decrease in mean CFU counts	
	AS1934	-	-	-	-	-	Exhibited a stronger decrease in mean CFU counts	
Pseudaminic acid metabolism (Pse)	CD23703	-	-	-	-	-	-	77
	CD09463	-	-	-	-	-	-	
	CD36508	-	-	-	-	-	-	
	CD26389	-	-	-	-	-	-	
	CD24868	-	-	-	-	-	-	

Protein	Compound name / ID	Cytotoxicity assays				*In vivo* assays		Reference
		Cell line	LC_{50} (μM)	TC_{50}	IC_{50} (μM)	Animal model	Effect on Colonization	
β Sliding Clamp	5-chloroisatin	-	-	-	-	-	-	78
	3,4-difluorobenzamide	-	-	-	-	-	-	
Thioredoxin reductase (TrxR)	2	-	-	-	Between 40 and 60	-	-	79
	3	-	-	-	Between 40 and 60	-	-	
	4	-	-	-	18.7	-	-	
	5	-	-	-	10.1	-	-	
	6	-	-	-	Between 60 and 80	-	-	
Thioredoxin reductase (TrxR)	3c	HEK-293 T	-	29.3 ± 1.6	-	-	-	80
	4a		-	50.2 ± 1.6	-	-	-	
	4c		-	20.7 ± 4.9	-	-	-	
	5a		-	58.0 ± 1.6	-	-	-	
	5c		-	30.2 ± 2.9	-	-	-	
Flavin-dependent thymidylate synthase (FDTS)	007-A (C8-C1)	Gastric adenocarcinoma	> 50 μg/mL	> 50 μg/mL	-	-	-	81

Protein	Compound name / ID	Cytotoxicity assays				*In vivo* assays		Reference
		Cell line	LC_{50} (μM)	TC_{50}	IC_{50} (μM)	Animal model	Effect on Colonization	
	010-C		> 50 μg/mL	> 50 μg/mL	-	Mice	Not significant	
	010-E		> 50 μg/mL	> 50 μg/mL	-		Not significant	
	010-I		> 50 μg/mL	> 50 μg/mL	-		17 fold decrease	
High-temperature requirement A Serine protease (HtrA)	1a	-	-	-	-	-	-	82
	1b	-	-	-	-	-	-	
	1c	-	-	-	-	-	-	
	2	-	-	-	-	-	-	
	5	-	-	-	-	-	-	
DL-carboxypeptidase (csd4)	Phosphinic acid 1	-	-	-	-	-	-	83
Cytotoxin-associated pathogenicity island protein 1 (CagA)	1G2	Gastric adenocarcinoma*	-	-	-	-	-	84
	1G2#1		-	-	-	-	-	
	1G2#2		-	-	-	-	-	
	1G2#3		-	-	-	-	-	
	1G2#4		-	-	-	-	-	

Protein	Compound name / ID	Cytotoxicity assays				*In vivo* assays		Reference
		Cell line	LC_{50} (μM)	TC_{50}	IC_{50} (μM)	Animal model	Effect on Colonization	
γ-Glutamyltranspeptidase (gGT)	AHPP-butyric acid (2-Amino-4[methyl (4-trifluoromethylphenyl) phospono]-butyric acid)	-	-	-	-	-	-	85
	AMOP-butyric acid (2-Amino-4-[methyl(4-methylumbelliferyl) phosphono]butyric acid)	-	-	-	-	-	-	
	GGsTopTM	-	-	-	-	-	-	
	DL-AP4-butyric acid (DL-2-Amino-4-phosphonobutyric acid)	-	-	-	-	-	-	
	Quisqualic acid		-	-	-	-	-	

Protein	Compound name / ID	Cytotoxicity assays				*In vivo* assays		Reference
		Cell line	LC_{50} (μM)	TC_{50}	IC_{50} (μM)	Animal model	Effect on Colonization	
Glucose-6-phosphate 1-dehydrogenase (G6PD)	YGC-1 scaffold 1,3-thiazolidine-2,4-dione	-	-	-	-	-	-	86
	MDG-1 1H-benzimidazole	-	-	-	-	-	-	
	MGD-2 1,3-benzoxazole	-	-	-	-	-	-	
	TDA-1 morpholine	-	-	-	-	-	-	
	JMM3 biphenylcarbonitrile	-	-	-	-	-	-	
Methionine/methionyl aminopeptidase (metAP)	7d	-	-	-	-	-	-	87
	7 e5	-	-	-	-	-	-	
	7d4	-	-	-	-	-	-	
	7g7	-	-	-	-	-	-	
	7e	-	-	-	-	-	-	
α-1,3-fucosyltransferase	3	-	-	-	-	-	-	88

Protein	Compound name / ID	Cytotoxicity assays				*In vivo* assays		Reference
		Cell line	LC_{50} (μM)	TC_{50}	IC_{50} (μM)	Animal model	Effect on Colonization	
Pyruvate ferredoxin oxidoreductase (PFOR)	Amixicile	-	-	-	-	-	-	89
	4.6	-	-	-	-	-	-	
	4.8	-	-	-	-	-	-	
	4.13	-	-	-	-	-	-	
	4.16	-	-	-	-	-	-	

Notes: * Molecules do not have negative impact on the viability of AGS cells at concentrations up to 500 μM

Abbreviations:

IC_{50} : Half maximal inhibitory concentration

LC_{50} : Median lethal concentration

TC_{50} : Toxic concentration 50

Table S3: Structural and Pharmacophoric Features of Non-Urease Targets in *Helicobacter pylori.*

Target	Dominant Scaffold / Chemical Class	Key Structural Requirements	Pharmacophoric Features	Ref.
Purine nucleoside phosphorylase (PNP)	2,6-disubstituted purines	Purine core is required for recognition; C2 and C6 substitutions modulate potency. A benzylthio group at C6 is associated with enhanced inhibition.	Hydrogen bond network: N7 forms hydrogen bonds with Asp204 and Ser203. π-stacking: The purine ring stacks with Phe159. Steric effects: Large C2 substituents (e.g., Cl) may displace Asp204, disrupting key interactions.	35-37
	Purine nucleoside core (C-type)	Requires a flexible scaffold to adapt to different active-site conformations (open/closed states).	Competitive inhibition: multiple binding modes (standard and nonstandard). Anchoring: critical interaction with Asp204; Phe159 contributes to proper substrate orientation.	
Adenylosuccinate synthetase (AdSS)	Aspartate-like scaffold	High structural similarity to L-aspartate; requires acidic groups (carboxylates) for recognition.	Substrate mimicry: competes for the aspartate-binding pocket. Characterized primarily by kinetic analysis (e.g., inhibition by hadacidin).	37-39
	Pyridoxal phosphate (PLP) scaffold	Presence of an aldehyde group for covalent engagement; structural features that mimic the GTP-binding region.	Covalent inhibition: formation of a Schiff base with Lys322 in the active site. GTP competition: mimics the interactions of the phosphate groups within the GTP-binding domain.	
Xanthine-guanine phosphoribosyltransferase (XGHPRT)	Purine phosphonate scaffold	Purine-mimetic core (Guanine/Xanthine type) for substrate recognition; Phosphonate group as a stable, non-hydrolyzable bioisostere of the natural phosphate.	Dual-site binding: simultaneous occupancy of the purine base-binding pocket and the phosphate-binding region. Metal coordination: Mg^{2+} is coordinated by both active-site residues (Asp/Glu) and phosphonate groups of the inhibitor.	40

Target	Dominant Scaffold / Chemical Class	Key Structural Requirements	Pharmacophoric Features	Ref.
Inosine-5'-monophosphate dehydrogenase (IMPDH)	Isoflavone scaffold (e.g., Irigenin)	Phenolic isoflavone framework derived from a natural product; hydroxylated polycyclic aromatic system.	Selective inhibition of *H. pylori* IMPDH over human IMPDH2; no structural binding data available; Represents a natural product–derived starting point for the development of selective HpIMPDH inhibitors.	41-47
	Sulfonyl amino acid + Anisamide	Sulfonyl amino acid derivatives linked to an anisamide scaffold; two aromatic rings connected by a linker; sulfonamide group attached to both aromatic moieties; amino acid side chain modulates activity and selectivity.	H-bond network: Consistent hydrogen bonding with Lys71 and Glu410. Additional anchoring via Ala246 for specific derivatives.	
	5-substituted Isobenzofuran-1(3H)-one	Phthalide core linked to a second aryl group via a short linker (2–3 atoms); linker length modulates activity and selectivity by optimizing hydrophobic and π interactions.	Aromatic interactions: π-stacking and hydrogen bonding within the cofactor-binding site.	
	3-aryldiazenyl indole	Indole core with an aryldiazenyl (N=N) system and acetamido linker; para-substituted electron-withdrawing groups must be avoided.	Non-competitive binding: likely targets a site distinct from the conserved IMP/NAD+ regions, reducing off-target effects.	
	Indole scaffold (alternative to benzimidazole)	Indole core introduced as an alternative to the benzimidazole scaffold; structural modifications aimed at improving selectivity toward bacterial IMPDH	Non-competitive inhibition with respect to IMP and NAD^+; molecular modeling suggests binding to a site distinct from the conserved IMP-binding region.	
	Chitosan Schiff base	Cross-linked chitosan backbone; functionalization with Schiff base groups and nanocomposites.	Polymeric Schiff base systems provide a platform for *H. pylori* IMPDH inhibition with combined antimicrobial functionality.	

Target	Dominant Scaffold / Chemical Class	Key Structural Requirements	Pharmacophoric Features	Ref.
	Methylpyrazole-substituted benzimidazole	Benzimidazole core modified with methylpyrazole; heterocyclic substitutions enhance potency over early leads.	Uncompetitive Inhibition: Binds to the E–S–C complex (IMPDH–IMP–NAD$^+$); stabilizes the ternary complex to prevent turnover.	
Shikimate kinase (SK)	Shikimic acid scaffold	Aromatic substituents at the C5 position; esterification (prodrug) to improve cellular permeability.	Hydrophobic interaction with a dynamic apolar pocket near C4–C5; stabilization of open LID and SB domain conformations; displacement of key arginine residues from the ATP-binding site.	49-51
	Bicyclic constrained shikimic acid	Conformational restriction via a C3–C5 ether bridge; pre-organizes the ligand into the bioactive geometry.	Enhanced multi-domain interactions (LID, SB, P-loop); stabilization via water-mediated interactions; improved binding through pre-organized conformations.	
	5-Aminoshikimic acid scaffold	Presence of bulky diarylmethyl groups at C5; reduced basicity of the amine is required to thrive in the apolar binding environment.	Hydrophobic engagement with a dynamic apolar gap in the SB domain (formed by helices α2, α3, and α5); stabilization of open LID and SB conformations, preventing active-site closure required for catalysis.	
Shikimate kinase (SK) / Urease / Aspartate-semialdehyde dehydrogenase	Diverse natural-product scaffolds (flavonoids, phenolic acids, glycosides)	Diverse phytochemical scaffolds identified through screening; no specific structural requirements explicitly established.	Putative binding to multiple enzyme targets suggested by molecular docking; no residue-level structural validation reported.	52
Shikimate dehydrogenase (SDH)	Amphipathic aromatic scaffolds	Aromatic core with oxygen-rich polar substituents (hydroxyl, carbonyl, and carboxyl groups); amphipathic balance is required for optimal pocket fit.	Subpocket selectivity: preference for targeting SP1 (Shikimate-binding) and SP2 (NADPH nicotinamide-binding) over SP3 (Adenine-binding). Stabilization via hydrogen bonding, π–π stacking, and van der Waals interactions	53

Target	Dominant Scaffold / Chemical Class	Key Structural Requirements	Pharmacophoric Features	Ref.
3-dehydroquinase dehydratase type II (DHQase II)	Quinazolinedione-based scaffold	Aromatic moiety tailored for the interface pocket; essential carboxylate group for hydrogen bonding; scaffold rigidification to limit catalytic loop movement.	Reversible competitive inhibition: binding at the active-site entrance stabilizes interactions with Tyr22, Arg17, and Asn76. Disruption of the substrate-covering loop positioning.	54
Aminodeoxyfutalosine nucleosidase (MTAN)	DADMe-Immucillin scaffold	Cationic hydroxypyrrolidine core (ribocation mimic); protonated N7 in 9-deazaadenine; mandatory 6-amino group.	Transition-state mimicry enabling tight binding; ion-pair interactions and active-site stabilization; extension into hydrophobic channels enhances affinity.	57-60
	5′-substituted DADMe-Immucillins	Variations in 5′-substituents tailored to fit the specific architecture of the bacterial hydrophobic tunnel.	Occupation of hydrophobic tunnel; binding affinity driven by complementarity with tunnel architecture; enables species-selective inhibition.	
	DADMe-Immucillin-A (MTDIA) with fragment extensions (TSID + FBDD)	Transition-state analogue core with fragment-based extensions (e.g., 2′-substituents); linker length and C4′ variation influence binding	Extension beyond canonical active site; engagement of additional binding regions (5′-region); enhanced affinity via expanded interaction network	
Aminofutalosine deaminase (AFLDA)	Coformycin-like scaffold	Mimics the catalytic intermediate (transition state); presence of a large pocket to accommodate 5′-substituents.	Metal coordination with active-site metal ion; hydrogen-bond network with catalytic residues stabilizes binding.	61
Aminodeoxyfutalosine/aminofutalosine synthase (MqnE)	Methylene substrate analogs	Structural mimics of the radical intermediate; replacement of the bridging oxygen with a methylene group (CH2) to block catalytic turnover.	Intermediate mimicry: mimics the captodative radical architecture. Tight-binding: Acts as a competitive inhibitor by preventing the formation of a stable reaction intermediate.	62
	Multivalent scaffolds	Combined architecture of two substrates into a single molecule to occupy multiple subpockets simultaneously.	Conformational sensitivity: binding affinity is significantly influenced by active-site flexibility; potency may be reduced if the linker prevents optimal orientation.	

Target	Dominant Scaffold / Chemical Class	Key Structural Requirements	Pharmacophoric Features	Ref.
Carbonic anhydrases α and β (αCA and βCA)	Phenolic scaffold (monoterpenoid phenols)	Small phenolic core with a critical hydroxyl group and hydrophobic substituents (methyl/isopropyl).	Zinc-water Interaction: phenols interact with the zinc-bound water/hydroxide. Negligible activity against human isoforms suggests a unique bacterial binding mode.	66-70
	Benzenesulfonamide scaffold (selenazole derivatives)	Benzenesulfonamide core linked to a selenazole heterocycle; diverse substitution patterns explored; small structural variations tolerated.	Binding within the carbonic anhydrase active site; consistent inhibitory activity across derivatives indicates limited sensitivity to structural variation (flat SAR)	
	Sulfonamide heterocyclic scaffold (thiadiazole-based)	Sulfonamide linked to heterocycle; substituents modulate orientation and binding.	Zn^{2+} coordination by sulfonamide nitrogen; transition-state mimicry of CO_2 hydration	
	Functionalized Coumarin Core	Coumarin core functionalized at C6/C7 with thiadiazole or triazolethione "tails."	Access channel occlusion: Likely acts via a prodrug mechanism where hydrolyzed species block the substrate access channel rather than direct metal binding.	
Glutamate racemase	Pyridodiazepine scaffold	Structural optimization focuses on balancing MurI potency with reduced plasma protein binding; requires specific physicochemical tuning to maintain inhibitory activity.	Substrate depletion: blocks D-Glu production, leading to the accumulation of UDP-MurNAc-Ala. Target engagement is confirmed by specific resistance mutations (A35T, A75T, C162Y).	71,72
	Natural-product-like (e.g., Bianthracenes like NP-020560)	Large, aromatic systems capable of occupying deep hydrophobic pockets; polar substituents required for hydrogen-bond stabilization.	π-stacking with Trp residues; hydrogen-bond network stabilizes allosteric binding.	

Target	Dominant Scaffold / Chemical Class	Key Structural Requirements	Pharmacophoric Features	Ref.
NADH-quinone oxidoreductase subunit D	Thienopyrimidine scaffold	C4 side-chain optimization enhances potency; α-carbon branching (R-enantiomer) preferred over β-carbon; hydroxyl group is essential for activity; phenyl substitutions are tolerated; position-dependent substitution on the core with C6 accommodating bulky groups and C5 being sterically restricted.	Critical hydrogen bonding: retention of a hydroxyl group is mandatory for anchoring to Thr400 (a known resistance residue). Interface binding: Targets the pocket at the NuoB–NuoD subunit interface.	73
Bifunctional enzyme IspD/IspF	Diverse Heterocyclic Scaffolds (e.g., Pyrimidine/Pyrazine diones, Quinolones)	Chemically diverse cores; polar functional groups are required to engage the enzyme's substrate-binding region.	Binding to substrate-binding regions of IspD domain; inhibition correlates with pathway disruption.	74,75
	Rosmarinic Acid	Polyphenolic structure with multiple hydroxyl groups; high hydrogen-bonding capacity is the primary driver of affinity.	Extensive H-Bond network: coordinates with a specific residue cluster: Arg108, Arg110, Gln111, Asp130, Arg133, and Lys202.	
	Tanshinone derivatives	Fused tricyclic abietane-type skeleton; more rigid and less polar than rosmarinic acid.	Hydrophobic/polar balance: shows more limited interaction capacity compared to phenolics; binding strength is dictated by the fit within the hydrophobic regions of the pocket.	
Dihydroorotate dehydrogenase (DHODH)	Quinolone-based scaffold (intervenolin derivatives)	Intervenolin core with modifications in pendant substituents; substitution of imines with dithiocarbamates to enhance stability.	Binding disrupts pyrimidine biosynthesis; active-site interaction inferred.	76

Target	Dominant Scaffold / Chemical Class	Key Structural Requirements	Pharmacophoric Features	Ref.
Pseudaminic acid metabolism (Pse)	Pyrrole / Pyrrolidone core	Common substructure designed to fit the deep UDP-sugar binding pocket. Substitution at the phenyl ring modulates orientation.	Substrate mimicry: occupies the cleft normally reserved for UDP-sugar pyrophosphate. Hydrophobic tuning: increased lipophilicity is required to penetrate the Gram-negative cell envelope.	77
	Diverse heterocyclic cores	Polar functional groups are positioned to interact with the sugar-binding subpockets; solvent-exposed groups modulate solubility.	Active site cleft binding: blocks the catalytic machinery required for dehydratase activity. Inhibits the biosynthesis of pseudaminic acid, leading to flagella loss.	
β Sliding Clamp	Diverse small-molecule scaffolds (e.g., 5-chloroisatin, carprofen, 3,4-difluorobenzamide)	Low-molecular-weight chemotypes identified from screening; predominantly hydrophobic scaffolds adapted to fit a conserved protein–protein interaction (PPI) cleft.	Binding within subsite I of the PPI cleft; predominantly hydrophobic interactions with key residues (Thr173/Thr175, Ile248, Pro243, Met370); limited hydrogen bonding (e.g., Thr175) contributes to stabilization and competition with protein partners.	78
Thioredoxin reductase (TrxR)	GlcNAc-thiosugar–Au(I)	Central Au(I) moiety; GlcNAc-based ligand with N-acyl substitution at C-2; acetylation state at C-3/C-4/C-5 modulates polarity.	Covalent inhibition: Au(I) center interacts with redox-active thiol (Cys) residues, disrupting the cellular redox balance. Phosphine substituents (Me vs. Et) dictate lipophilicity.	79, 80
	Au(I)–NHC scaffold	Replacement of the phosphine ligand with N-heterocyclic carbenes (NHC); NHC substituents are variable; thioglucose is non-essential for some derivatives.	Interaction with redox-active thiols; enzyme inhibition via metal coordination.	
Flavin-dependent thymidylate synthase (FDTS)	2-Hydroxy-1,4-naphthoquinone	The 2-OH group is essential for catalytic interference; substituents are optimized to balance nanomolar potency with reduced mitotoxicity.	Active site blockade: binds within the substrate pocket, directly overlapping the dUMP-binding region. Inhibits the oxidation of NADPH.	81

Target	Dominant Scaffold / Chemical Class	Key Structural Requirements	Pharmacophoric Features	Ref.
High-temperature requirement A Serine protease (HtrA)	Fragment-derived aromatic scaffold	Aromatic moieties (phenyl-furanyl region) coupled with carboxylic acid groups; removal of reactive functional groups to prevent aggregation and improve metabolic stability.	Thiol-redox disruption: confirmed inhibition of TrxR. The NHC ligand provides superior electronic stability and lowers toxicity compared to PEt3.	82
DL-carboxypeptidase (csd4)	Phosphinic acid pseudodipeptide	Phosphinic acid replaces the natural iso-D-Glu–meso-Dap amide bond; reproduces the geometry of the tetrahedral oxyanion intermediate.	Bidentate coordination with Zn^{2+}; interaction with catalytic residues.	83
Cytotoxin-associated pathogenicity island protein 1 (CagA)	Diverse fragment scaffolds	Small fragments with variable chemotypes; no unified pharmacophores.	Binding outside active site; conformational modulation of protein assembly.	84
γ-Glutamyltranspeptidase (gGT)	Diverse scaffolds (electrophilic inhibitors and amino acid–like compounds)	Distinct heterocyclic rings tailored to fit the $NADP^+$ binding region; scaffold geometry modulates the depth of pocket penetration. Linear or branched aromatic systems that exploit the hydrophobic regions surrounding the catalytic core.	Active-site binding through two interaction modes: covalent modification of catalytic Thr380 by electrophilic inhibitors; non-covalent interactions with glutamate-recognition regions for reversible inhibitors	85
Glucose-6-phosphate 1-dehydrogenase (G6PD)	Heterocyclic scaffolds (thiazolidinedione, benzimidazole, benzoxazole)	Core scaffolds adapted to $NADP^+$ binding region.	$NADP^+$ mimicry: acts as a competitive inhibitor by occupying the cofactor catalytic site. Mixed inhibition: modeling suggests secondary binding sites that enable non-competitive/uncompetitive behavior relative to G6P.	86
Methionine/methionyl aminopeptidase (metAP)	Azaindole–hydroxamic acid scaffold	An azaindole core linked to a hydroxamic acid metal-binding group (MBG); structural variations are specifically tailored to fit the S1 pocket.	Metal coordination: the hydroxamic acid moiety coordinates the catalytic metal ions (Co^{2+} or Mn^{2+}). Pocket selectivity: interactions with "left lobe" residues in the S1 pocket.	87

Target	Dominant Scaffold / Chemical Class	Key Structural Requirements	Pharmacophoric Features	Ref.
α-1,3-fucosyltransferase	GDP-triazole derivatives	GDP moiety is mandatory for anchoring; a triazole linker mimics/extends the pyrophosphate bridge; pendant sugars (fucose, galactose) modulate affinity.	Donor-binding site competition: acts as a competitive inhibitor against the natural donor substrate (GDP-fucose). Acetylation of the sugar moiety can increase potency.	88
Pyruvate ferredoxin oxidoreductase (PFOR)	Amixicile (Nitrothiazole-benzene-amine)	Three-zone design: (i) Nitrothiazole head; (ii) Benzene linker; (iii) Variable amine tail. The nitro group is mandatory for TPP interaction.	Cofactor interference: interacts with Thiamine Pyrophosphate (TPP). The nitro group facilitates proton abstraction from the TPP aminopyrimidine.	89
